# Impact of short-duration voltage variations on VSC-HVDC performance

**DOI:** 10.1038/s41598-023-50362-3

**Published:** 2023-12-27

**Authors:** Reem A. Mostafa, Adel Emary, A. Sayed, M. EL-Shimy

**Affiliations:** 1 Electrical Power and Machines Engineering Department, Faculty of Engineering, Ain Shams University, Cairo, Egypt; 2 National Energy Control Center –Egyptian Electricity Transmission Company, Cairo, Egypt; 3 Electrical Power and Machines Engineering Department, High Institute of Engineering, El Shorouk City, Cairo Egypt

**Keywords:** Electrical and electronic engineering, Energy infrastructure

## Abstract

The growing load demand globally necessitates increasing the penetration of renewable energy sources into electrical grids as well as interconnecting grids from different countries and even continents through HVDC transmission systems. Since these applications rely on power electronics devices, several power quality issues arise, namely voltage sags and swells. This paper analyzes the response of a VSC-HVDC transmission system that interconnects two asynchronous AC grids to short-duration voltage variations like sag and swell by adjusting the voltage of the controllable AC source. The system is simulated with the help of MATLAB/Simulink. The study records the effect of the manipulated AC voltage on the active/reactive powers and AC/DC voltages at both converter stations to evaluate the system stability due to these prevalent power quality challenges. The obtained results reveal that the system hardly withstands voltage variation for a short period.

## Introduction

Power quality (PQ) phenomena have lately received great attention due to the wide utilization of sensitive nonlinear power electronic devices in various fields, like industrial applications, and long-distance electrical power transmission. In addition, the penetration of renewable energy sources (RES) into the power grid has grown, which results in new PQ challenges, especially short-duration voltage variations (sag and swell), as they are frequently occurring. That is why analyzing the characteristics, causes, and consequences of different PQ disturbances becomes vital^1–4^.

High voltage direct current (HVDC) transmission system is an appropriate choice to link remote RES with electrical grids, and directly interconnect asynchronous AC grids as well^5–10^. It provides grid stability, robust operation, power transmission over long distances, high efficiency, and slight power losses^11–14^. Recent research has comprehensively reviewed the voltage-sourced converter-based HVDC (VSC-HVDC) type focusing on its available topologies and control methodologies^4,9,15^. It properly facilitates the integration of RES into conventional power generation^3,16–18^. In addition, the VSC-HVDC topology has been frequently employed in long-distance power transmission since it has favorable PQ characteristics, and efficient performance^19,20^. It is barely vulnerable to commutation failures; hence, it is applied in multi-terminal HVDC systems known as “Super Grid”^14,21^.

Although PQ disturbances have been extensively studied in the electrical power system field, it is one of the challenges for the proposed super grids which rely on the VSC-HVDC transmission system due to connecting new generation technologies to the AC terminals of this enormous grid^22^. For example, the connection of photovoltaic (PV) panels to the AC side, at a low voltage level, causes overvoltage and the switching frequency of wind turbine (WT) converters results in injecting high-frequency waveforms continuously into the grid. Furthermore, the load fluctuations at the AC receiving end cause intrinsic PQ problems like voltage dips and swells. Recent research has not adequately addressed the study of voltage control and stability under these disturbances^4,15,19^.

This study specifically examines the response of VSC-HVDC to short-duration voltage variations like sag and swell. It accomplishes this through the determination of two critical parameters: the critical clearing time (CCT) and the critical voltage (CV). These parameters provide an essential indication to the system's ability to remain stable after being subjected to such disturbances. These perturbations are applied to the system as step inputs to the adjustable AC source.

The paper begins with a brief introduction to the importance of studying the PQ challenges in VSC-HVDC applications. The available topologies are then discussed, with a focus on the most appropriate one. Additionally, the layout of the VSC-HVDC transmission system linking two asynchronous AC systems is presented. The vector control strategy at both converter stations is discussed in detail, including the available control modes at each station. The system is then simulated using MATLAB Simulink under sag and swell disturbances in both stable and unstable cases. Furthermore, analyzing the consequences of these PQ challenges is performed by observing the active/reactive power and the AC/DC voltage waveforms of both converter stations. Additionally, plotting the trajectories of the active versus reactive power (P-Q) and reactive power versus RMS AC voltage (Q–U) at each converter station is a critical step in assessing the system's ability to regain stability after being subjected to these power quality (PQ) issues. Finally, the results of each case study are comprehensively discussed.

### Overview of VSC-HVDC transmission technology

**Figure 1:**
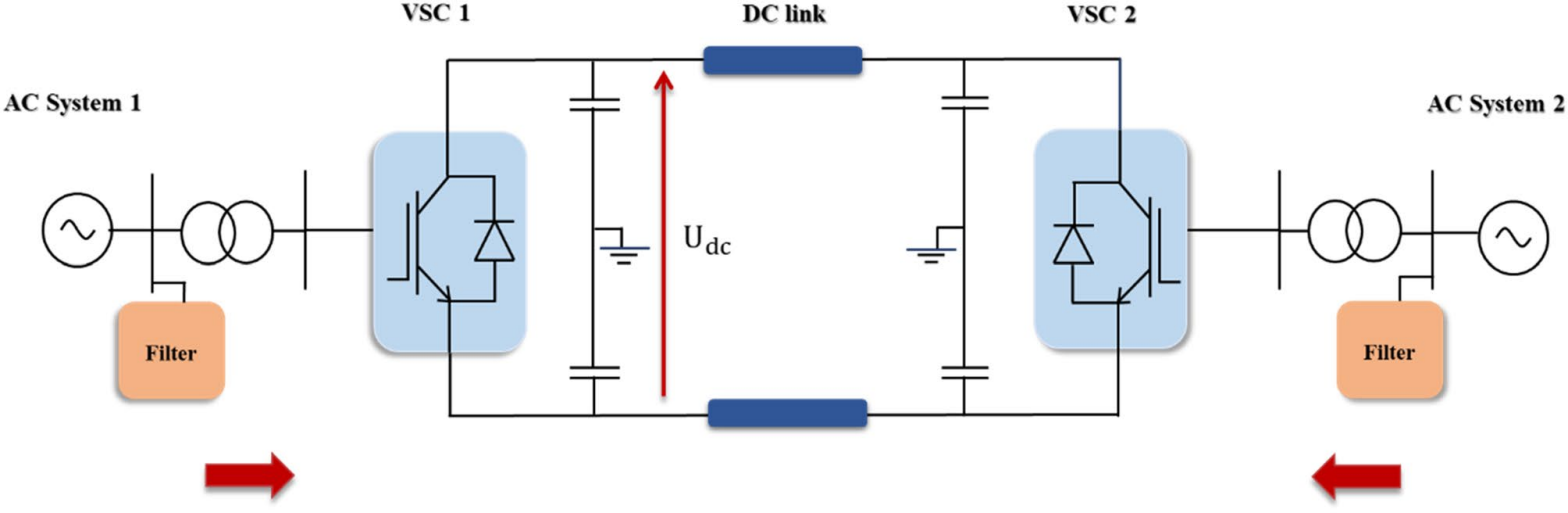
VSC-HVDC transmission layout directly connects two AC systems.

VSC-HVDC is a highly controllable transmission topology whose principal function is continuously transmitting constant electrical power from one AC system to the other. The typical layout of the VSC-HVDC system connecting two AC sources is illustrated in Fig. 1^8,16,23^.

As depicted in Fig. 1, the network primarily comprises two AC networks where the power is transmitted. Both AC grids are simulated as a three-phase voltage source connected to a transformer to step down the AC voltage to an appropriate value that the converter station can withstand. In addition, they are equipped with AC filters to avoid the harmonic impact on the AC network performance^16,23^.

Then, two VSC stations are employed to transmit the electrical power through a DC link; one of them acts as a rectifier and the other station as an inverter according to the power flow. Both converters usually rely on IGBT/Diodes in the form of a two-level, six-pulse bridge, or a three-level, 12-pulse bridge. With the help of pulse width modulation (PWM) methodology, the voltage waveform can be adjusted instantaneously^16,23,24^. Finally, two identical capacitor banks are utilized at each DC side, as shown in Fig. 1, to provide an electrostatic energy storage device and minimize the voltage ripples as well^8,16,23^.

### Prevalent power quality issues in HVDC transmission systems

**Figure 2:**
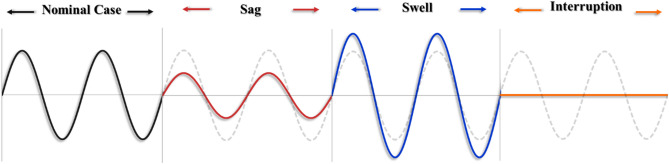
Common short-duration voltage variations impacting HVDC systems.

As declared by IEEE Standard^25^, PQ problems are categorized based on the deviation of the magnitude of the nominal value as well as the duration for which the disturbance lasts until it is eliminated. According to the duration, PQ issues can be classified into short and long-duration disturbances. Voltage imbalance, power frequency variations, transient and waveform distortion are also considered in this classification. Since HVDC systems primarily rely on employing power electronics devices, they are frequently impacted by PQ problems, especially short-duration voltage variations, namely sags, swells, and interruptions that last for less than one minute, as stated in IEEE Standard^25^. As a result, the linked AC systems’ performance is affected. Figure 2 shows the difference between these PQ issues.

Short-duration voltage sag is the most prevalent PQ phenomenon that can be characterized by the reduction in RMS voltage at the power frequency (50 Hz) for 3:30 cycles. In HVDC transmission systems, it frequently results from numerous AC faults, namely single-line-to-ground, line-to-line, and double-line-to-ground faults that occur in AC power system equipment. In addition, DC faults such as pole-ground, pole-to-pole, and double-pole-to-ground occurring in the DC transmission link can cause voltage dips^23,26^. As the occurred fault draws a high current, it consequently causes a significant voltage drop at fault point^27–29^. Furthermore, the unbalanced load at one of the AC sides may result in undesirable voltage fluctuations. Sags have severe consequences that seriously impact the connected loads at each AC terminal. Power electronic components as well as control devices like adjustable-speed drive (ADS) will stop. Consequently, the operation of industrial loads such as motors will be interrupted. Furthermore, the delivered electrical energy to the consumer will be significantly reduced.

Another common PQ problem that may appear in HVDC transmission systems is the short-duration voltage swell. In contrast to a sag disturbance, it refers to a rise in the RMS voltage magnitude at the power frequency (50 Hz) for 3:30 cycles. Swell is usually caused at one of the AC sides by disconnecting large loads such as motors, or wide areas (known as load shedding)^27^. Repetitive voltage swell may lead to insulation breakdown, and then conductors will be live in case of exceeding the acceptable swell limits.

As revealed in Fig. 2, instantaneous interruption is a special case of voltage sag that refers to the reduction of the supply voltage at one of the AC systems to less than 0.1 pu for 3:30 cycles based on 50 Hz power frequency^27^. It is usually caused by either a repetitive sag or an extended voltage sag. Its duration relies on the tripping time protection scheme used to protect the power system equipment. Such a disturbance may lead to a load outage that lasts for cycles^30^.

### VSC-HVDC constraints against power quality challenges

Despite the wide spread of VSC-HVDC methodology to transmit enormous power over long distances, it faces numerous PQ disturbances that inhibit its efficient performance especially when this transmission system is linked to a weak AC system. VSC stations are not capable of completely protecting the transmission system from voltage sags and swells caused by the AC terminals^31^. The control system of the converter station quickly recovers when the system is subjected to transient PQ disturbances like voltage spikes or dips at one of the AC sides. However, they are immune to the rapid recovery required for efficient mitigation of the prevalent PQ problems. To mitigate these issues, sophisticated control methodologies are required, considering the coordination of both the AC and DC sides. However, these PQ constraints can be mitigated in VSC-HVDC systems by utilizing additional equipment such as Flexible Alternating Current Transmission Systems (FACTS) namely static synchronous compensator (STATCOM)^32,33^, energy storage devices like superconducting magnetic energy storage (SMES)^34^, and using advanced control algorithms^34^. Furthermore, HVDC transmission should be properly designed, considering the nature of the interconnected AC systems as well as the connected loads to ensure that the PQ problems are overcome.

## Simulation and mathematical model

**Figure 3:**
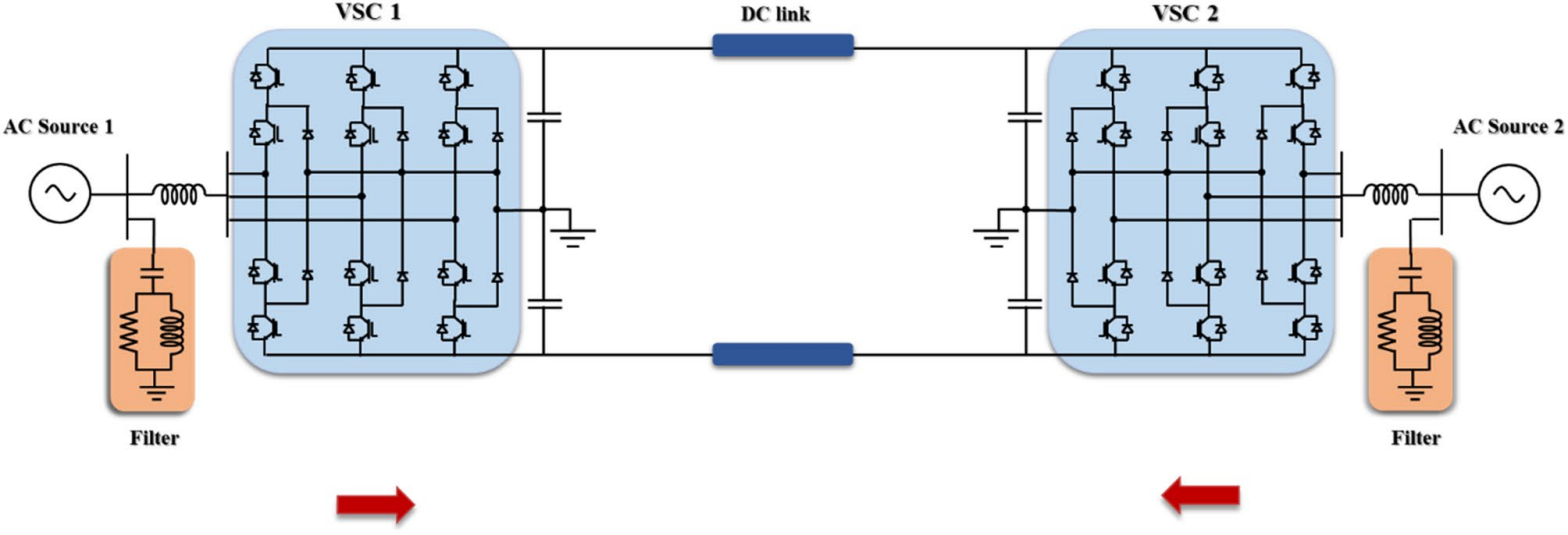
Model of VSC-HVDC transmission system interconnecting two AC systems with different frequencies.

**Figure 4:**
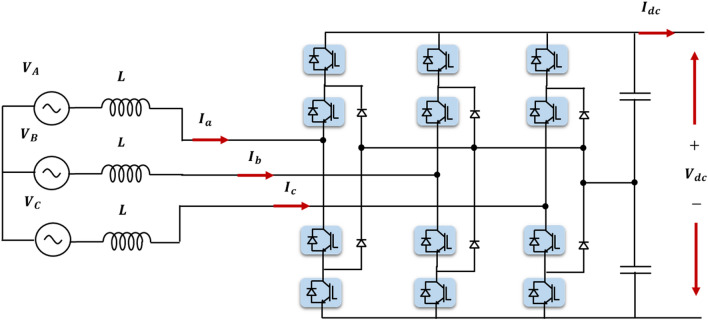
A three-level NPC-based VSC connected to a three-phase AC source.

Figure 3 represents the modified MATLAB model of a VSC-HVDC transmission system. The main function of this network is interconnecting two asynchronous AC sources considering the HVDC system as the transmission link where the model data are stated in Table 1. As shown in Fig. 3, each AC system is connected to the HVDC system through a VSC station (VSC_1_ and VSC_2_). Both converter stations are identical utilizing a three-level Neutral Point Clamped (NPC) converter (see Fig. 4). It relies on insulated gate bipolar transistors (IGBT)/diodes because of their favorable characteristics^21^. They are primarily used in industrial applications as they produce negligible harmonics and reduce the losses to approximately 1.7%^35–37^. Since the power flow is bidirectional, one of the converter stations acts as a rectifier and the other one acts as an inverter. However, the simulation is conducted under the assumption that VSC_1_ and VSC_2_ interface the sending and receiving ends respectively during the steady state.

**Table 1:** Parameters of VSC-HVDC transmission system connecting two AC sources differing in frequency^38^.

Component	Parameter	Value
AC Source 1	RMS AC voltage of the AC source (kV)	230
	AC source frequency (Hz)	50
	Apparent power of the transformer (MVA)	200
	Transformer ratio Y/Δ (kV)	230/100
AC Source 2	RMS AC voltage of the AC source (kV)	230
	AC source frequency (Hz)	60
	Apparent power of the transformer (MVA)	200
	Transformer ratio Y/Δ (kV)	230/100
DC transmission line	Number of pi sections	2
	Line length (km)	75
	Resistance (mΩ/km)	13.9
	Inductance (μH/km)	159
	Capacitance per (nC/km)	231
VSC1&2	Converter type	NPC utilizing IGBT/Diodes
	Number of arms	3
	DC voltage (kV)	100

**Figure 5:**
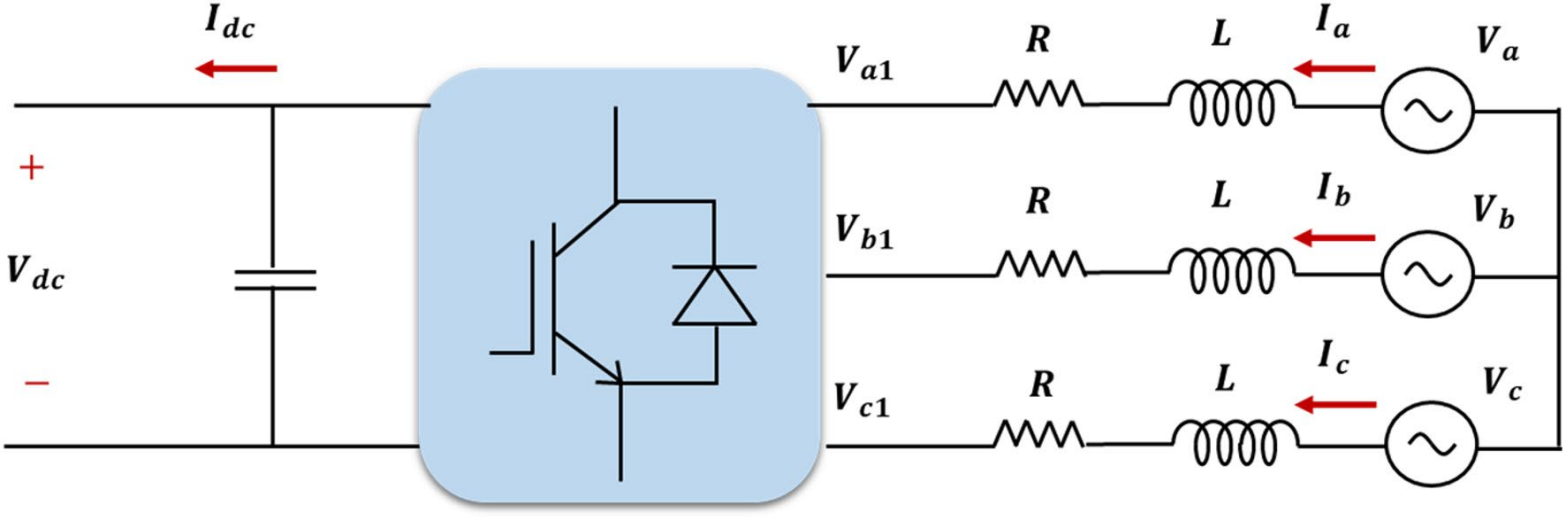
The equivalent circuit of a VSC station connected to a three-phase AC source.

Figure 5 illustrates a simplified circuit that connects a VSC station to a three-phase AC source. In addition, the relation between the AC voltage at the converter terminal, the AC source voltage, and the produced current at each phase is given in (1)^39^.

1 $$\left\{\begin{array}{c}{V}_{a}={V}_{a1}+{i}_{a}{R}_{a}+j\omega L\frac{d{i}_{a}}{dt}\\ {V}_{b}={V}_{b1}+{i}_{b}{R}_{b}+j\omega L\frac{d{i}_{b}}{dt}\\ {V}_{c}={V}_{c1}+{i}_{c}{R}_{c}+j\omega L\frac{d{i}_{c}}{dt}\end{array}\right.$$

**Figure 6:**
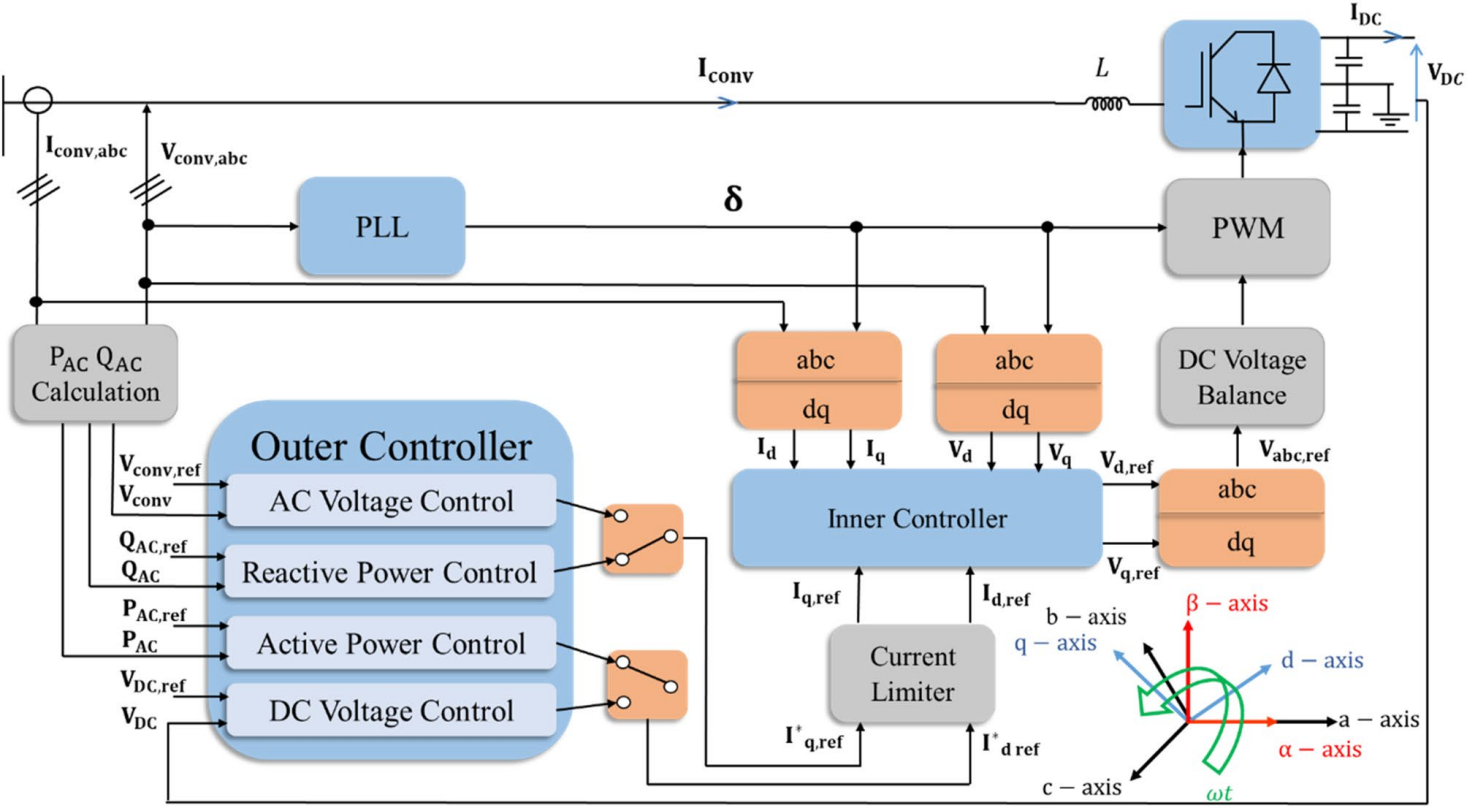
Operational principle of the vector control strategy for a VSC station.

The control scheme of the VSC-HVDC system is shown in Fig. 6. It provides control of the active/reactive power as well as the DC voltage at each converter station. As shown in Fig. 3, the AC side of each VSC station is connected to an AC source via a reactance; hence, the active and reactive power of the converter can be calculated using Eqs. (2) and (3) where the harmonics and reactance losses are neglected^8,21,40^.

2 $${P}_{VSC}= \frac{{V}_{s}\mathit{sin}(\delta )}{X} {V}_{c}$$

3 $${Q}_{VSC}= \frac{{V}_{s}\mathit{cos}\left(\delta \right)-{V}_{c}}{X} {V}_{c}$$

where Vs is the AC source voltage, Vc is the AC voltage at the converter, X is the inductive reactance of the reactor, and δ is the phase shift between the two voltages Vs and Vc.

The most common technology used in VSC applications is DQ control. This method depends on transforming the stationary three-phase AC voltages and currents into a rotating dq domain according to (4)^41–43^. The preferred control mode is chosen by the outer control loop. The direct component of the current I_d_ determines whether DC voltage or active power is to be controlled, whereas the quadrature component I_q_ selects either reactive power or AC voltage to be controlled. The outputs of this loop are the reference direct and quadrature components of the current I_d,ref_, I_q,ref_, respectively, which are the inputs of the inner control loop. It is responsible for producing the reference voltage waveforms V_ref_ required for the pulse width modulation (PWM) of the converter. The phase-locked loop (PLL) determines the reference angle of Park’s transformation performed in the inner current loop. In addition, it aligns the d-axis with the voltage at the point of common coupling (PCC). The active/reactive converter current control as well as the PWM methodology cause minor variation at the DC side of the converter station. As a result, applying the DC voltage balance control becomes essential to ensure the balance of the DC link during the steady state, as demonstrated in Fig. 6^10,42,44^.

4 $$\left[ {\begin{array}{*{20}c} {V_{d} } \\ {V_{q} } \\ {V_{0} } \\ \end{array} } \right] = \frac{2}{3} \left[ {\begin{array}{*{20}c} {\sin (\omega t)} & {\sin (\omega t - \frac{2\pi }{3})} & {\sin (\omega t + \frac{2\pi }{3})} \\ {\cos (\omega t)} & {\cos (\omega t - \frac{2\pi }{3})} & {\cos (\omega t + \frac{2\pi }{3})} \\ \frac{1}{2} & \frac{1}{2} & \frac{1}{2} \\ \end{array} } \right]\left[ {\begin{array}{*{20}c} {V_{a} } \\ {V_{b} } \\ {V_{c} } \\ \end{array} } \right]$$

## Case studies

**Figure 7:**
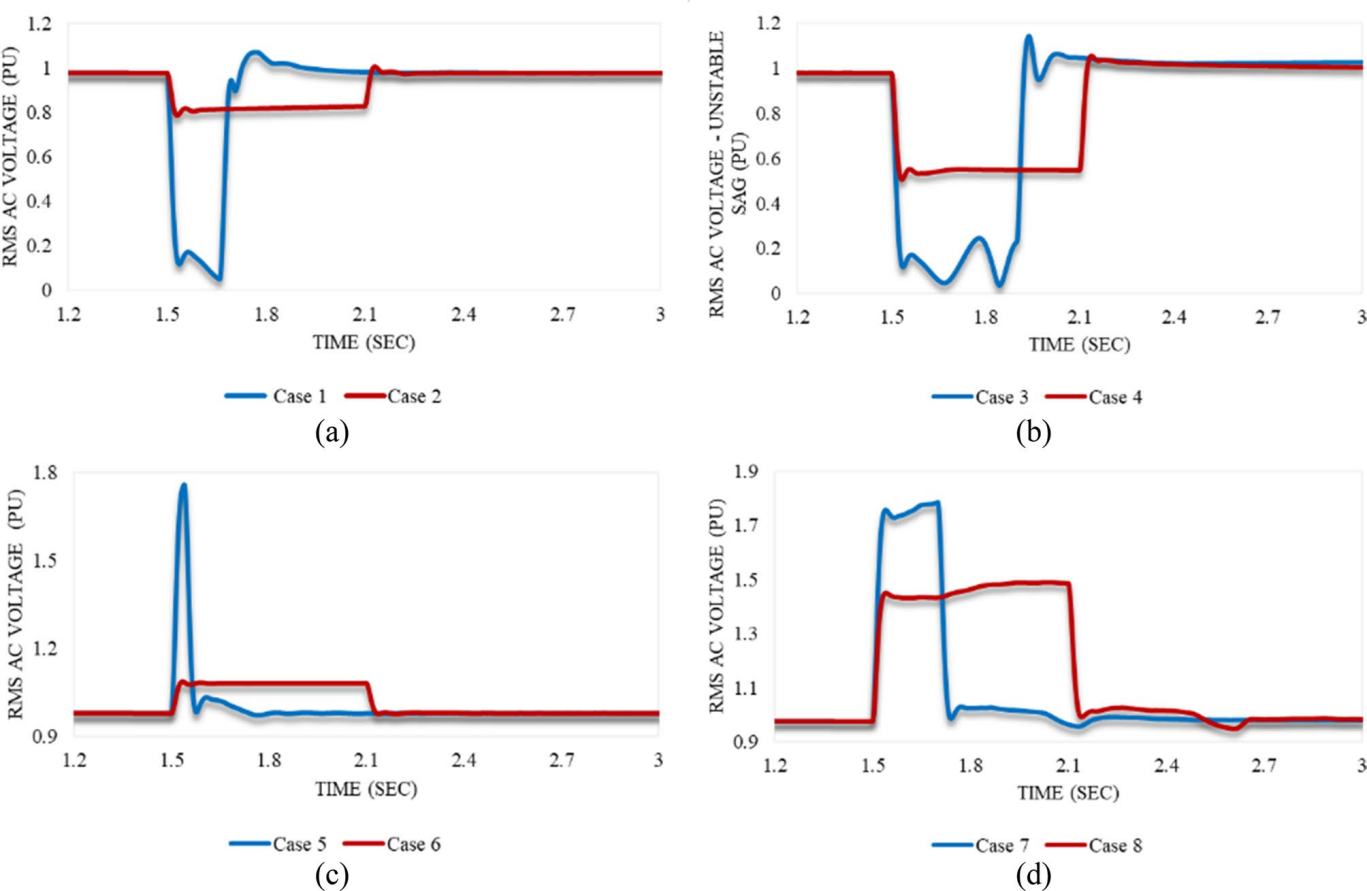
Model response after applied voltage variations at 1.5 s: (**a**) stable voltage sags (case 1 and case 2), (**b**) unstable voltage sags (case 3 and case 4), (**c**) stable voltage swells (case 5 and case 6), and (**d**) unstable voltage swells (case 7 and case 8).

Numerous PQ issues, namely sag and swell are applied to this model by adjusting the voltage of the controllable AC source at VSC_1_. For each disturbance, the system is studied in both stable and unstable conditions. Figure 7 depicts all the PQ issues that are applied to the model in this study. In the case of a stable system, the CV magnitude after which the system will hardly attain its stability is determined. The CCT is also deduced. Active/reactive powers and AC/DC voltage waveforms at both converter stations are recorded and analyzed. In addition, the trajectories of the active/reactive powers and reactive power/AC voltage at each station are figured out. Table 2 summarizes the maximum permissible limits for either the voltage magnitude or the duration, known as the critical conditions of the applied disturbance (sag/swell).

**Table 2:** The maximum permissible limits for sag and swell disturbances.

Acceptable limits	Magnitude (pu)	Duration (cycles)
Instantaneous voltage sag	0.9	8
	0.2	30
Instantaneous voltage swell	0.8	2
	0.1	30

The critical values of the instantaneous voltage sag are determined. Based on the model frequency (50 Hz), the system will successfully attain its steady state when it is applied to the max sag (0.9 pu) for 8 cycles “Case 1”. In contrast, it can withstand instantaneous sag for its maximum interval (30 cycles) when it is impacted by 0.2 pu sag “Case 2”. To simulate an unstable sag, the system will be studied after being applied to a disturbance that causes it to barely regain the steady state. This can be achieved by exceeding the critical limits for either the interval (for case 1) or the amplitude (for case 2). According to Fig. 7, the system is subjected to a sag disturbance of 0.9 pu at 1.5 s for 20 cycles “Case 3”, and 0.5 pu at 1.5 s for 30 cycles “Case 4”.

In addition, the critical values of the instantaneous voltage swell are deduced. The system can successfully regain the steady state when it is subjected to the maximum swell (0.8 pu) for 2 cycles only “Case 5” based on the model frequency which is 50 Hz. However, it can withstand the instantaneous swell for its maximum interval (30 cycles) when it is affected by 0.1 pu swell “Case 6”. For unstable swell simulation, the system is impacted by a swell disturbance so that the steady-state condition is hardly recaptured. This may result from exceeding the critical limits for either the interval (for case 5) or the amplitude (for case 6). According to Fig. 7, the system is subjected to a swell disturbance of 0.8 pu at 1.5 s for 10 cycles “Case 7”, and 0.5 pu at 1.5 s for 30 cycles “Case 8”.

## Discussion of the case studies’ results

### Impacting the model with stable sag disturbances: Case 1 & Case 2

**Figure 8:**
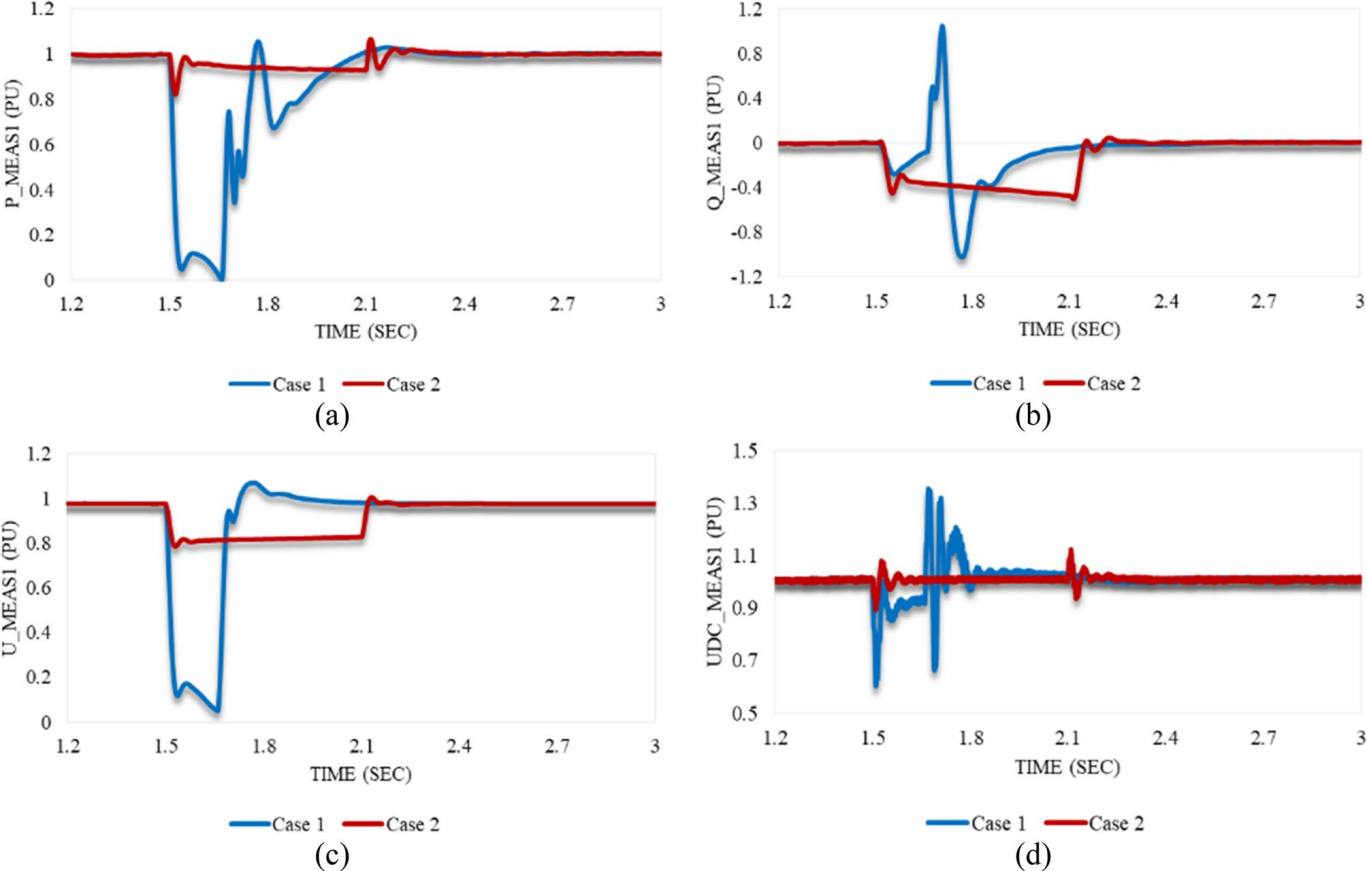
Measured waveforms at VSC_1_ when a stable sag disturbance is applied to the model at 1.5 s in both cases 1 and 2: (**a**) the active power, (**b**) the reactive power, (**c**) the RMS AC voltage, and (**d**) the DC voltage.

The response of P_meas1 in both cases 1 and 2 is shown in Fig. 8a. It simultaneously follows up the applied disturbance in case 1 by a great reduction up to zero, then gradually regains its stability at 2.056 s. In contrast, P_meas1 decreases to a US% of 17.9 just for an instant when the disturbance of case 2 is subjected to the model. It attains a steady state through two stages. It decreases slightly, then suddenly rises at 2.1 s above the reference value. Finally, it becomes stable at 2.108 s. This significant decay indicates the reverse power direction; hence, VSC_1_ draws active power from the other AC side during this temporary perturbation.

Since the applied voltage dip reduces the RMS AC voltage at VSC_1_, the reactive power demand should increase to compensate for this sudden decay. Furthermore, it is observed that the reactive power in case 2 is less susceptible to voltage reduction. As can be seen in Fig. 8b, Q_meas1 responds with a slight reduction, followed by a great OS% of 104.8 in case 1. It regains its stability at 1.9 s. The response of Q_meas1 in case 2 counteracts what occurred in case 1. It significantly reduces at the instance at which the disturbance is applied, then slightly increases. Afterward, it continuously decreases to a US% of 49.97. It becomes stable again at 2.25 s.

Figure 8c reveals that the behavior of U_meas1 in cases 1 and 2 is the same. It keeps declining, reaching an undershoot, then quickly recapturing its stability. However, it is noticed that the US% of case 1 is much greater than case 2. In addition, U_meas1 recovers the steady state in case 1 quicker than in case 2. As depicted in Fig. 8d, Udc_meas1 declines to a US% of 39.64 at 1.51 s in case 1, but it reaches an OS% of 12.4 at 2.1 s in case 2. Udc_meas1 returns to the steady state at 1.8 s in case 1. In contrast, stability is recovered at 2.14 s in case 2.

**Figure 9:**
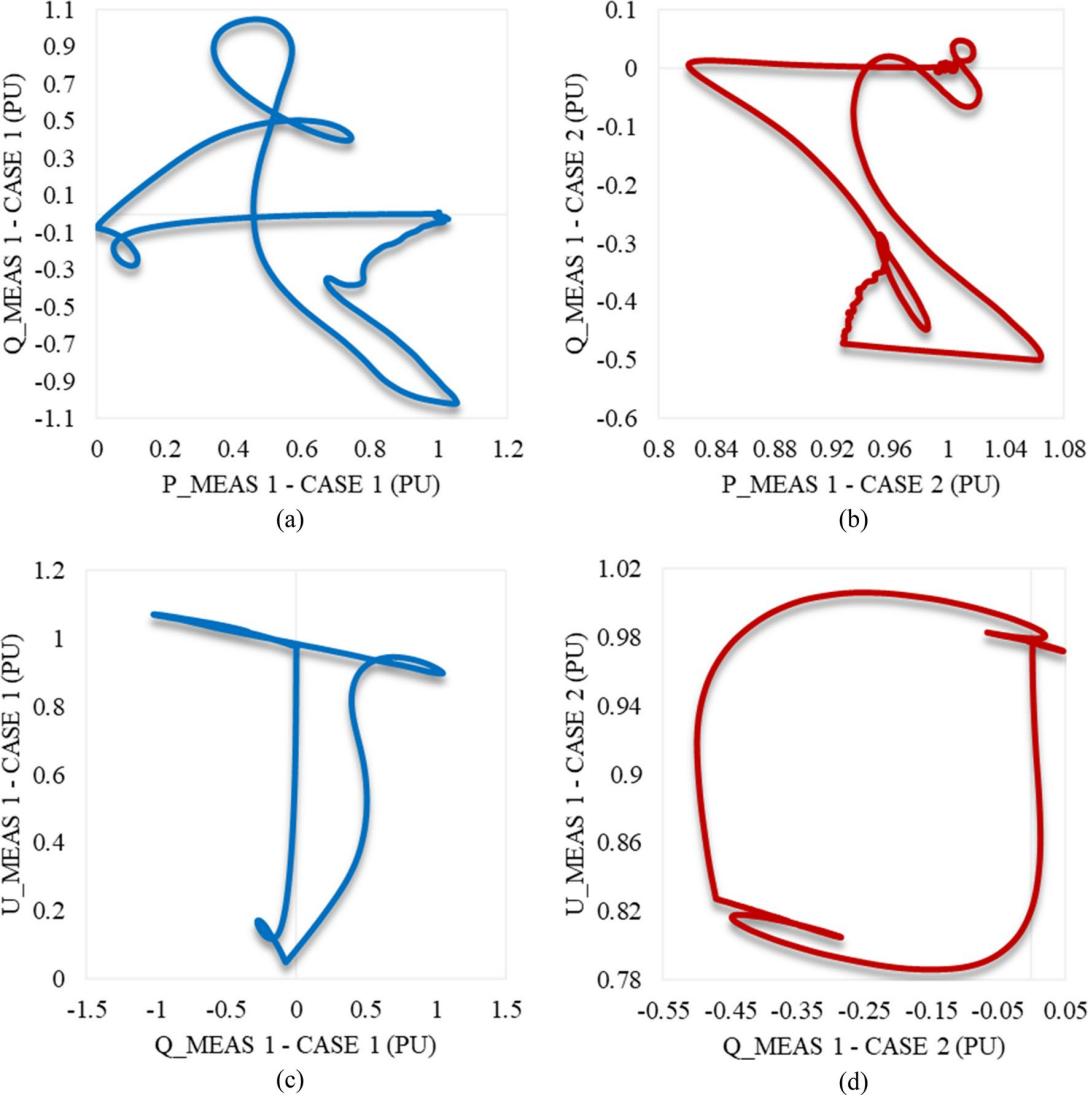
Trajectories of (**a**) active/reactive power at VSC_1_ in case 1, (**b**) active/reactive power at VSC_1_ in case 2, (**c**) reactive power/RMS AC voltage at VSC_1_ in case 1, and (**d**) reactive power/RMS AC voltage at VSC_1_ in case 2.

Figure 9a,b represent the trajectory of P_meas1 and Q_meas1, explaining their responses in both cases 1 and 2, respectively. The trajectory of Q_meas1 and U_meas1 in cases 1 and 2 is illustrated in Fig. 9c,d respectively. Since the trajectories converge to a fixed point in both case studies, that indicates system stability. It will eventually reach a balanced operational condition.

**Figure 10:**
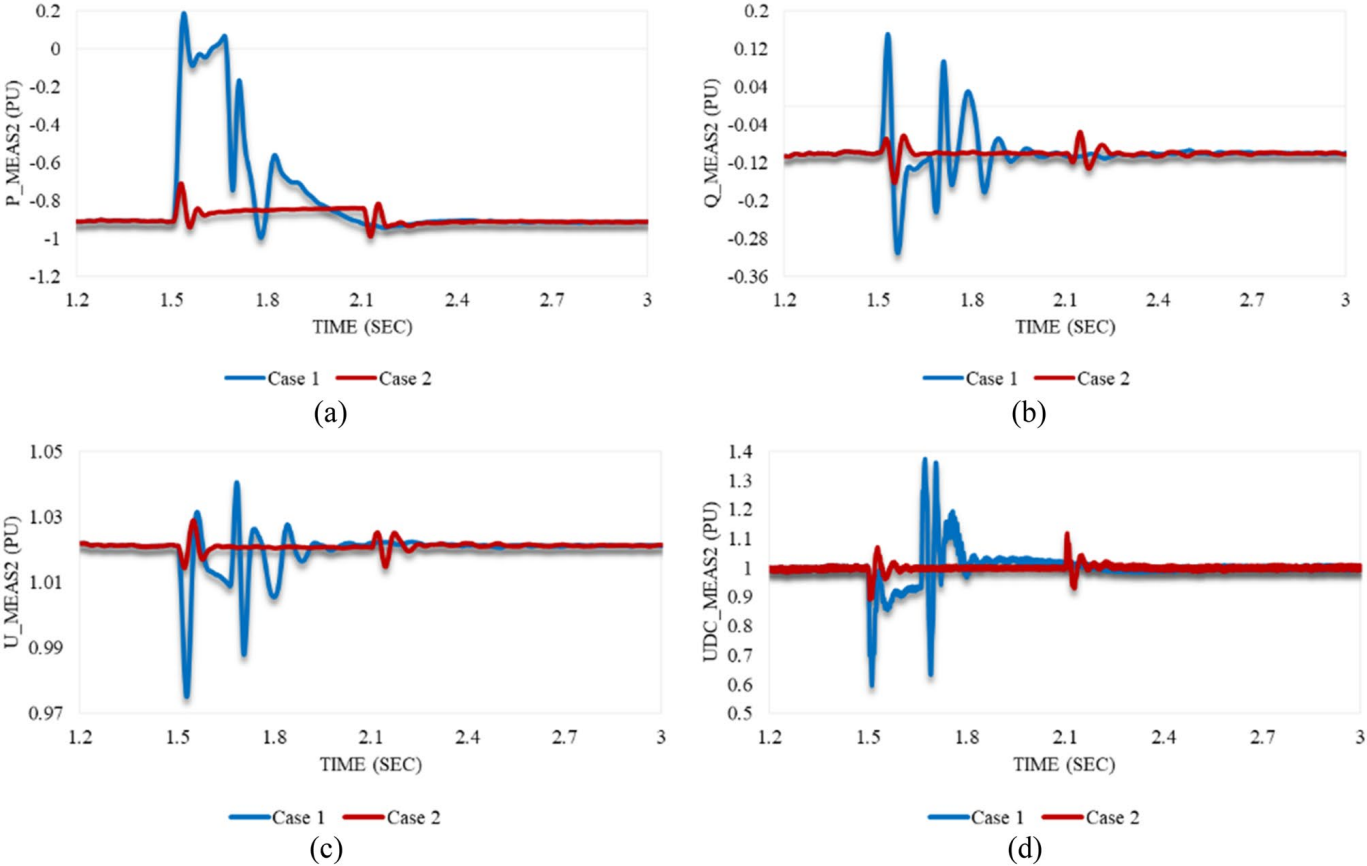
Measured waveforms at VSC_2_ when a stable sag disturbance is applied to the model at 1.5 s in both cases 1 and 2: (**a**) the active power, (**b**) the reactive power, (**c**) the RMS AC voltage, and (**d**) the DC voltage.

The behavior of P_meas2 counteracts the response of P_meas1 in either case 1 or 2 since it becomes the sending end temporarily. As depicted in Fig. 10a, P_meas2 grows steeply to an OS% of 120.6 immediately when the disturbance of case 1 is applied to the system. Then, it declines with oscillating amplitude until it reaches the steady state at 2.06 s. In contrast to case 1, P_meas2 reaches an OS% of 20% for an instance. It slowly oscillates until it recovers the stability conditions.

As can be seen in Fig. 10c, the RMS AC voltage at VSC_2_ is indirectly affected by the imposed disturbance on VSC_1_. U_meas2 slightly reduces compared to the behavior of U_meas1 in either case 1 or 2. As a result, the reactive power at VSC_2_ simultaneously increases to follow up this response as shown in Fig. 10b. Q_meas2 steeply rises, then keeps oscillating until the stability condition is reached. However, Q_meas2 responds to case 2 with a significant reduction followed by oscillations. At 2.13 s, it suddenly increases and oscillates until it attains system stability. Both Figs. 8-d and 10-d indicate the consistent behavior of the DC voltage at both stations, irrespective of the magnitude or duration of the sag applied to the system. The DC voltage remains unaffected, demonstrating a resilient response in maintaining its steady-state value throughout the system.

**Figure 11:**
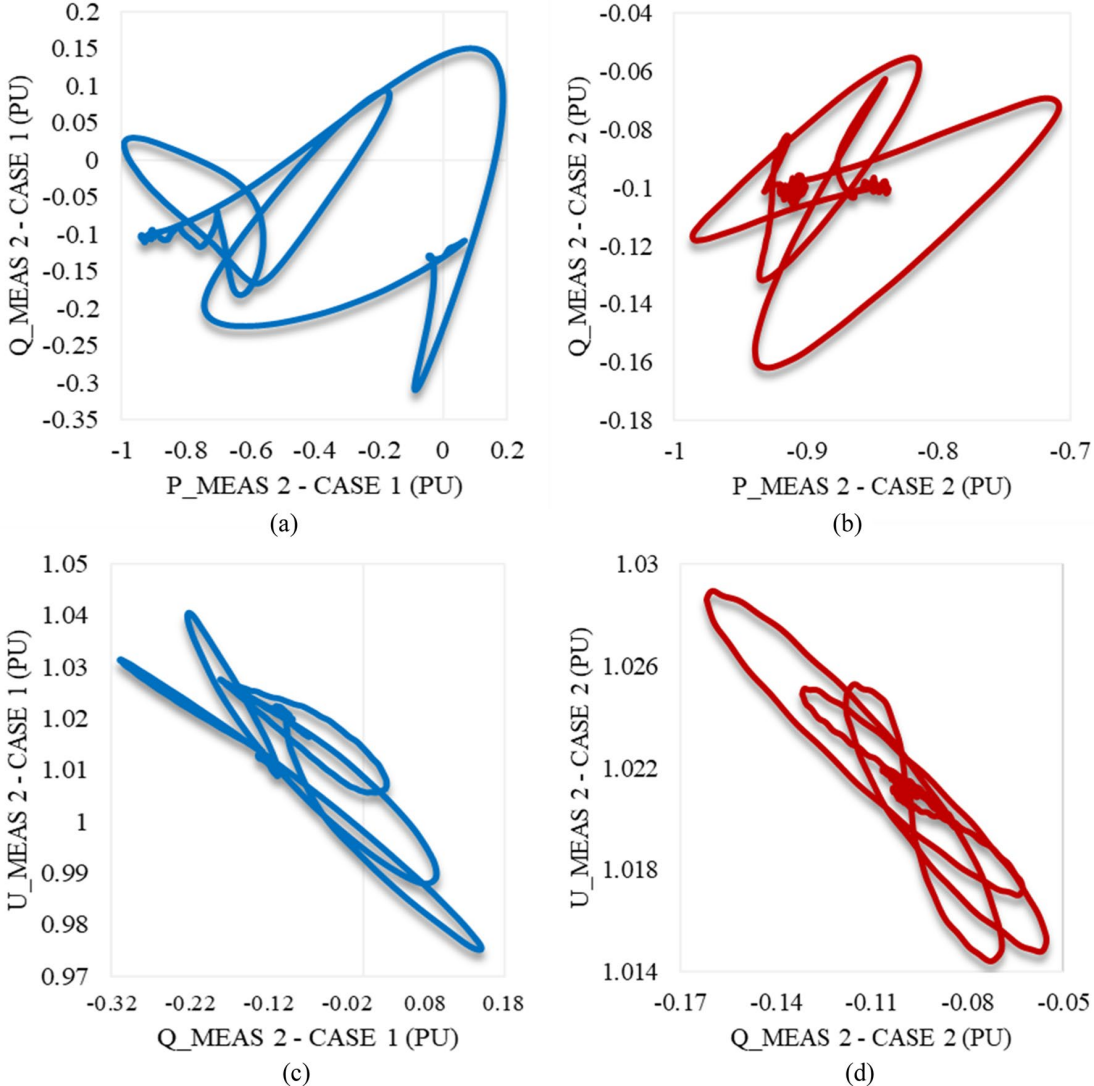
Trajectories of (**a**) active/reactive power at VSC_2_ in case 1, (**b**) active/reactive power at VSC_2_ in case 2, (**c**) reactive power/RMS AC voltage at VSC_2_ in case 1, and (**d**) reactive power/RMS AC voltage at VSC_2_ in case 2.

The trajectory of P_meas2 and Q_meas2 that explains their response in both cases 1 and 2 is illustrated in Fig. 11a,b respectively. Figure 11c,d depict the trajectory of Q_meas2 and U_meas2 in case 1 and case 2 respectively as well. The closed paths of trajectories in Figs. 9 and 11 refer to the capability of converter stations 1 and 2 respectively to regain stability if the system is subjected suddenly to the maximum sag limits (0.9 pu) just for 8 cycles based on a system frequency of 50 Hz. In addition, the system is also able to recover the steady state in case of being applied to only 0.2 pu sag and lasts for the maximum duration of the instantaneous disturbance (30 cycles based on a frequency of 50 Hz).

### Impacting the model with unstable sag disturbances: Case 3 & Case 4

**Figure 12:**
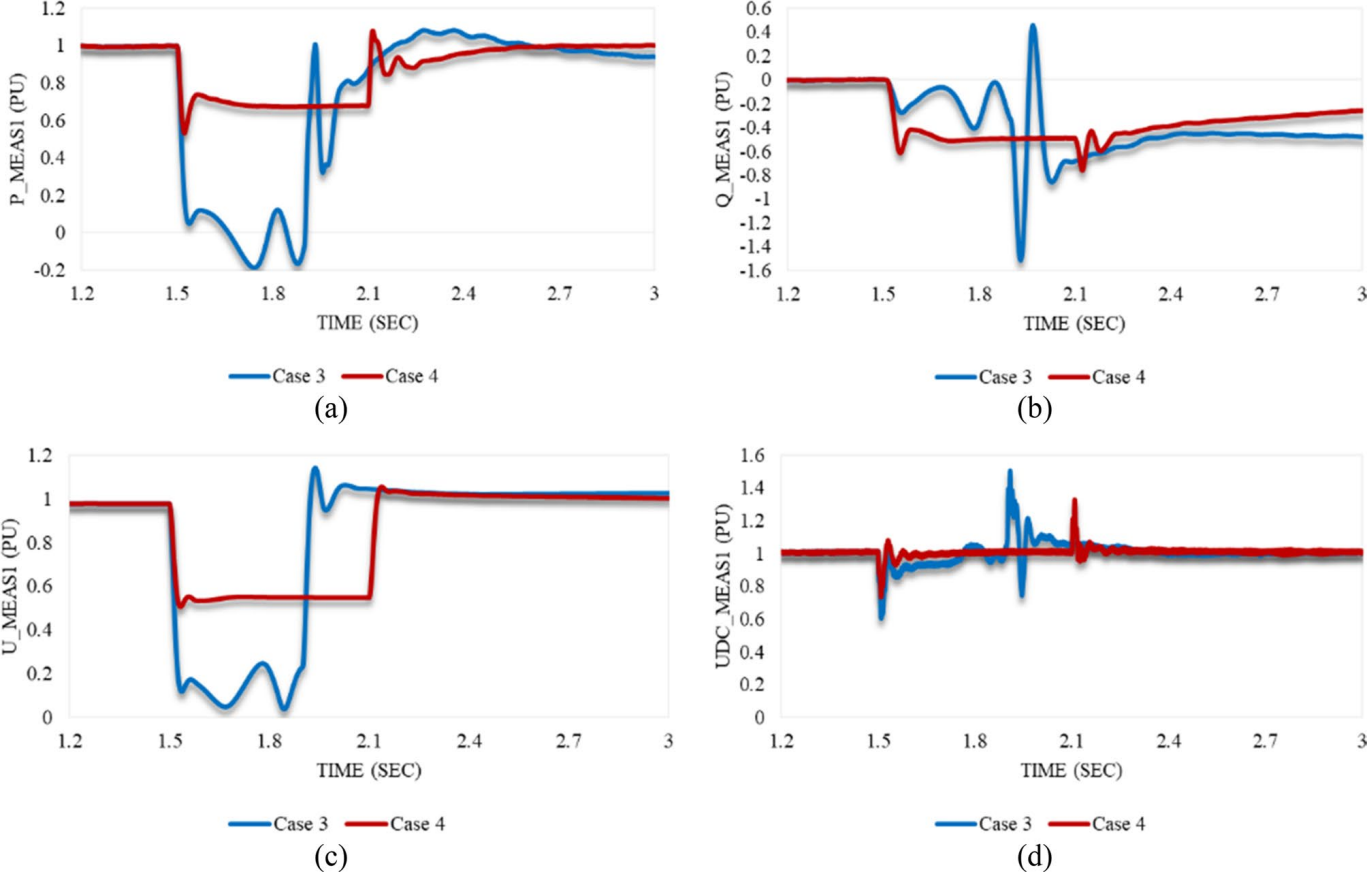
Measured waveforms at VSC_1_ when an unstable sag disturbance is applied to the model at 1.5 s in both cases 3 and 4: (**a**) the active power, (**b**) the reactive power, (**c**) the RMS AC voltage, and (**d**) the DC voltage.

When the system is impacted by the disturbance in case 3, P_meas1 declines simultaneously. After 0.33 s from the instance at which the disturbance is applied to the system, a gradual increase is noticed until reaching a virtual steady state with an SSE of -0.06 pu (see Fig. 12a). In contrast, it temporarily decreases and remains constant until 2.1 s. Then, it oscillates until attains a steady state with a negligible SSE of 0.002 pu. This response reveals the simultaneous decay of the transmitted active power between the two converter stations due to the severe sag conditions, which prevent the system from attaining a steady state once again.

The behavior of Q_meas1 in both cases 3 and 4 is illustrated in Fig. 12b. The drawn reactive power by VSC_1_ greatly rises in both cases to overcome the sudden voltage reduction. In case 3, Q_meas1 responds by gradual reduction till attaining a significant US of 150%, followed by a steep rise. It reaches a steady state with a significant SSE of -0.47 pu. As depicted in Fig. 12a,b, the Q_meas1 response is similar to P_meas1 in case 4, except for the permanent instability of Q_meas1 with an SSE of -0.2 pu.

As can be seen from Fig. 12c, U_meas1 is affected by case 3 through a significant US then it remains oscillating with an SSE of 0.048 pu. Figure 12c also reveals that the behavior of U_meas1 in both cases 3 and 4 is the same. First, it falls to an undershoot then recaptures its stability. Despite the great US% of case 3 compared to case 4, U_meas1 recovers the steady state in case 3 quicker than case 4. The response of the DC voltage waveforms at both stations is the same. Despite the system instability in cases 3 and 4, it fluctuates until reaching the steady state as shown in Figs. 12d and 14d.

**Figure 13:**
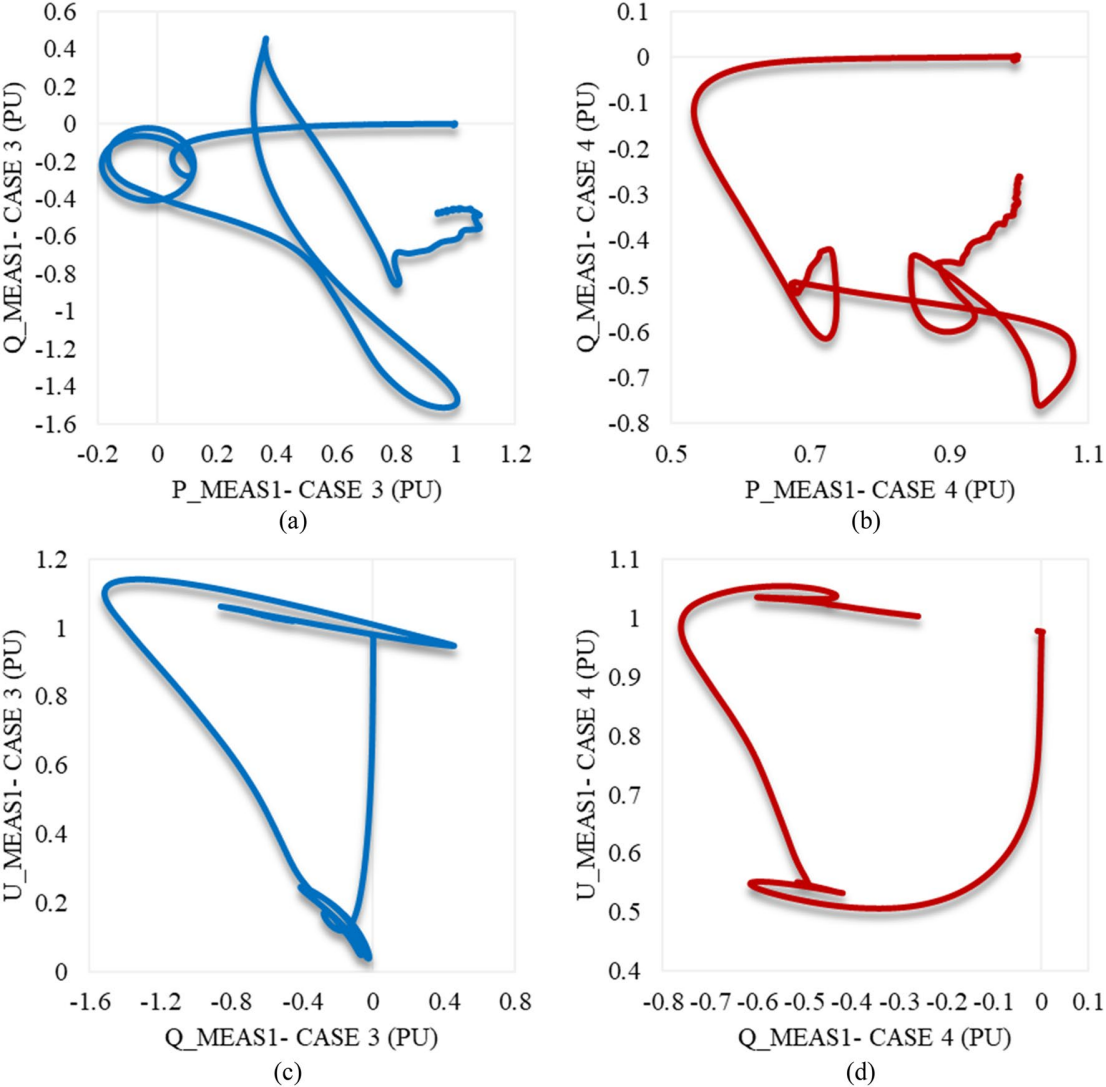
Trajectories of (**a**) active/reactive power at VSC_1_ in case 3, (**b**) active/reactive power at VSC_1_ in case 4, (**c**) reactive power/RMS AC voltage at VSC_1_ in case 3, and (**d**) reactive power/RMS AC voltage at VSC_1_ in case 4.

Trajectories of P_meas1/Q_meas1 illustrating their responses to each other in cases 3 and 4 are shown in Fig. 13a,b respectively. In addition, Fig. 13c,d represents the trajectories of Q_meas1/U_meas1 in cases 3 and 4 as well. According to Fig. 13, the starting point of each curve is far from the endpoint, which indicates system instability. It does not recapture the initial/reference conditions.

**Figure 14:**
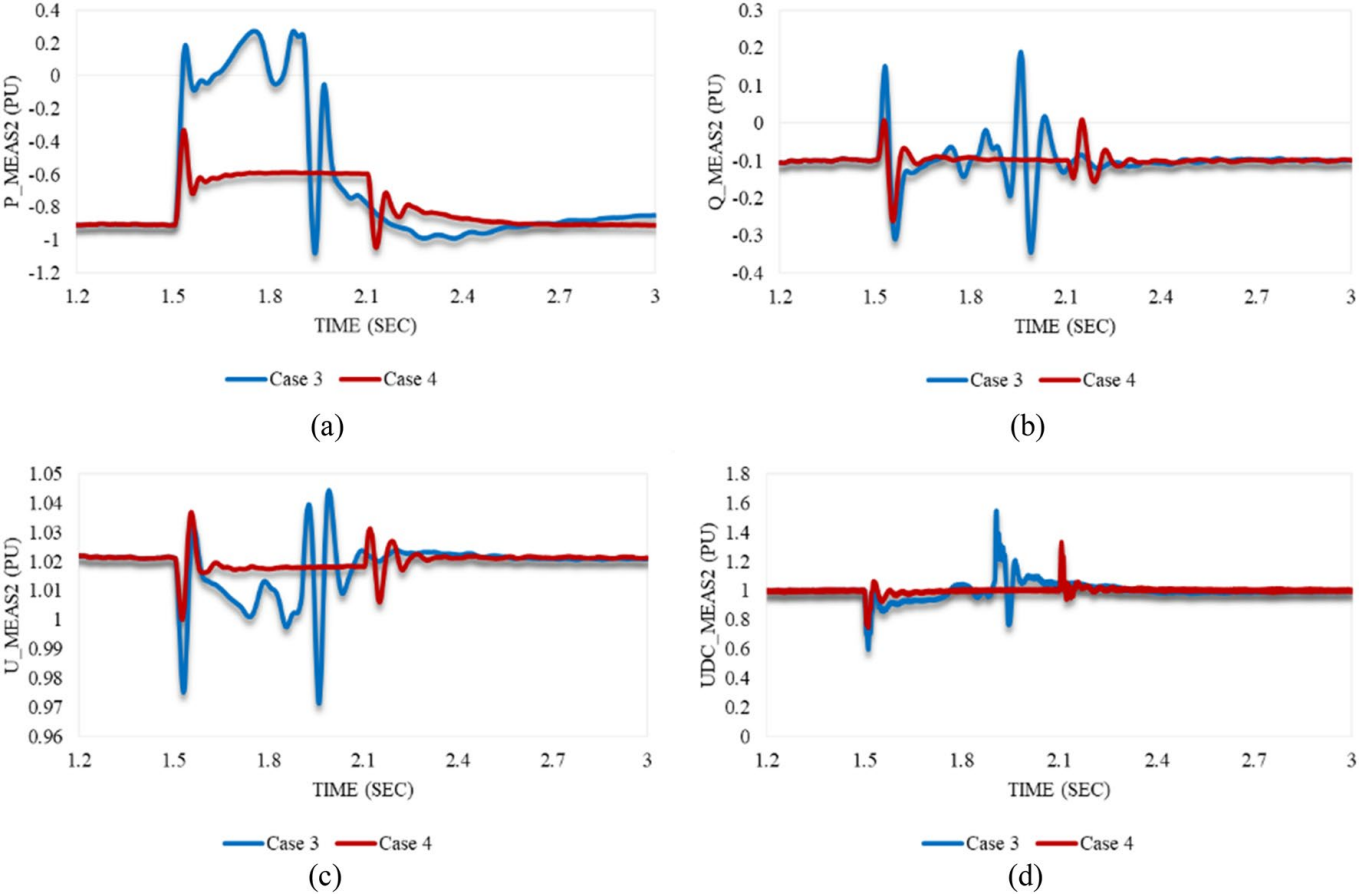
Measured waveforms at VSC_2_ when an unstable sag disturbance is applied to the model at 1.5 s in both cases 3 and 4: (**a**) the active power, (**b**) the reactive power, (**c**) the RMS AC voltage, and (**d**) the DC voltage.

**Figure 15:**
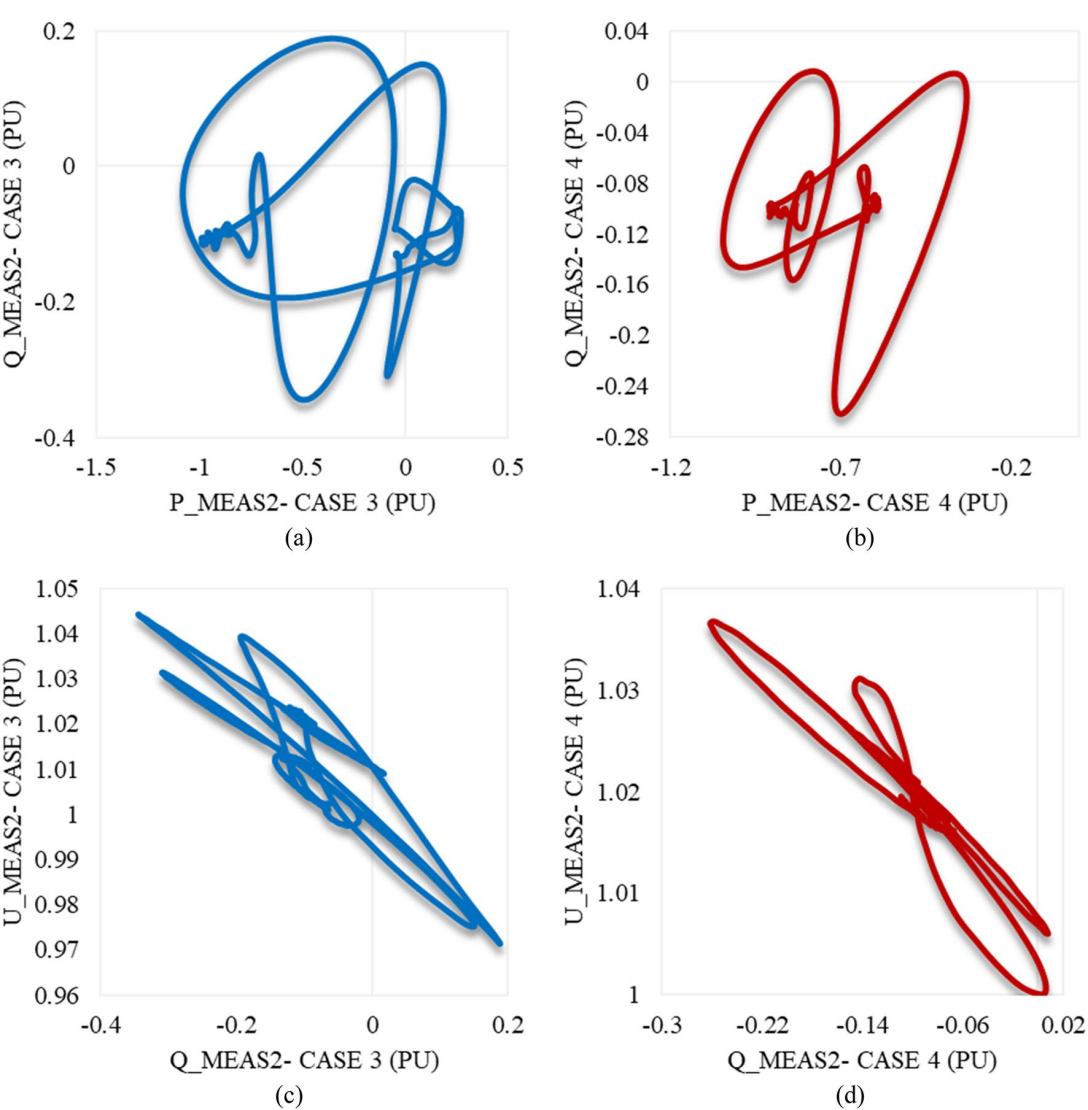
Trajectories of (**a**) active/reactive power at VSC_2_ in case 3, (**b**) active/reactive power at VSC_2_ in case 4, (**c**) reactive power/RMS AC voltage at VSC_2_ in case 3, and (**d**) reactive power/RMS AC voltage at VSC_2_ in case 4.

As depicted in Fig. 14a, P_meas2 responds equally to both disturbances in cases 3 and 4. It increases to supply the other converter station and then oscillates until reaching a steady state, except for the greater amplitude in case 3. The behaviour of Q_meas2 due to the applied disturbances in cases 3 and 4 is illustrated in Fig. 14b. It keeps oscillating until it attains the steady-state conditions, unlike the reactive power at station 1, which cannot regain stability. In contrast to the response of the AC voltage at station 1, U_meas2 responds with high-frequency oscillations and reaches the steady state with negligible SSE, which depicts that impacting one converter station by a voltage variation will affect the other for instance (see Fig. 14c). Figures 12d and 14d reveal that the DC voltage at both converter stations responds equally to these disturbances. Regardless of the disturbance amplitude or interval, the DC link voltage remains stable. According to Fig. 15a,b, the active/reactive power and AC/DC voltage at VSC_2_ cannot regain the steady state condition, which reveals the system instability due to the extreme sag conditions applied to the system at 1.5 s.

### Impacting the model with stable swell disturbances: Case 5 & Case 6

**Figure 16:**
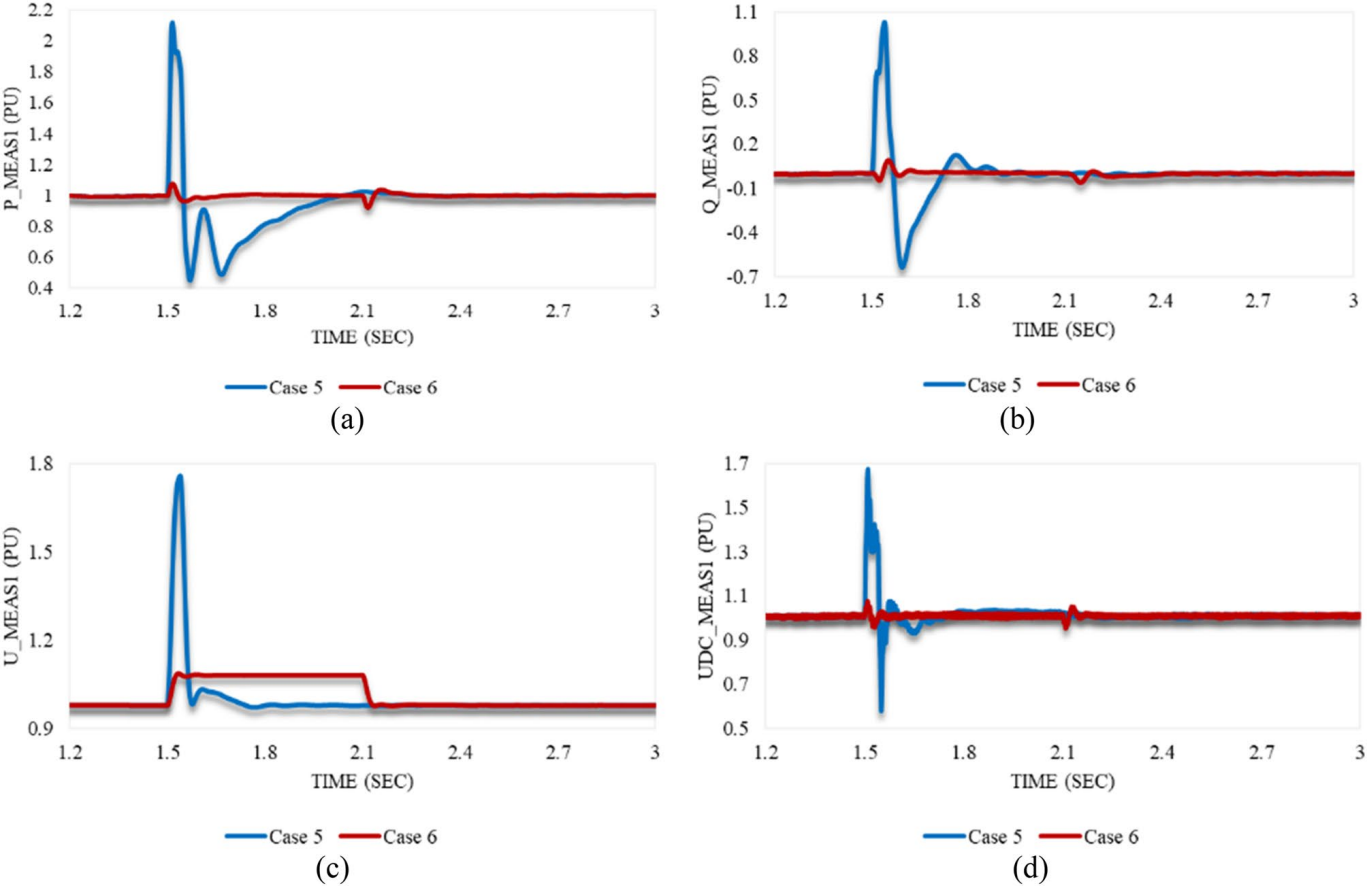
Measured waveforms at VSC_1_ when a stable swell disturbance is applied to the model at 1.5 s in both cases 5 and 6: (**a**) the active power, (**b**) the reactive power, (**c**) the RMS AC voltage, and (**d**) the DC voltage.

Unlike the voltage sag case studies, the increased RMS voltage at VSC_1_ due to the applied swell results in a great rise in the transmitted active power from VSC_1_ to VSC_2_. Figure 16a illustrates the response of P_meas1 in both cases 5 and 6. In case 5, it grows simultaneously with the disturbance till reaching an OS% of 111.8 followed by a steep reduction. It then increases gradually until the stability is regained at tsett = 1.99 s. In contrast, the behavior in case 6 is slight. P_meas1 rises to a small overshoot before temporarily reaching the steady state. At 2.1 s, it reduces until it stabilizes at tsett = 2.14 s.

As shown in Fig. 16b, the reactive power at VSC_1_ grows in case 5 by 97.9% at 1.54 s. This excessive reactive power is supplied to VSC_1_. After that, a significant reduction is noticed until 1.6 s to overcome this sudden disturbance effect. It attains a steady state at 1.885 s. The behavior of Q_meas1 in case 6 is different from case 5. It repeatedly oscillates before reaching a steady state. Figure 16c reveals that the AC voltage at station 1 can regain the steady state condition in case of being subjected to either a voltage swell of 0.8 pu for 40 ms “Case 5” or a swell of 0.1 pu for 0.6 s “Case 6”. As shown in Fig. 16d, the DC voltage at station 1 oscillates with a higher amplitude in case 5 than in case 6. However, it stabilizes quickly in both cases.

**Figure 17:**
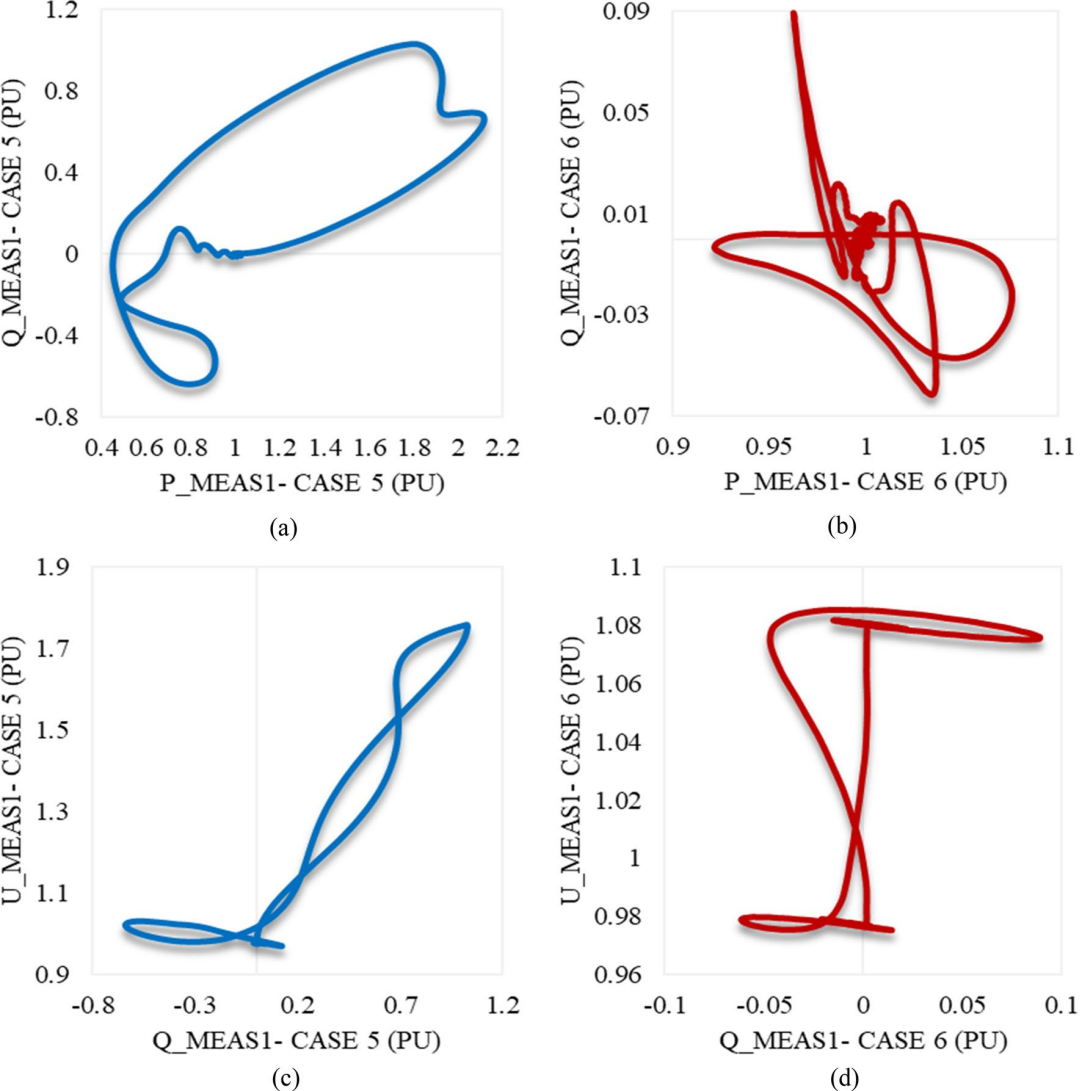
Trajectories of (**a**) active/reactive power at VSC_1_ in case 5, (**b**) active/reactive power at VSC_1_ in case 6, (**c**) reactive power/RMS AC voltage at VSC_1_ in case 5, and (**d**) reactive power/RMS AC voltage at VSC_1_ in case 6.

Figure 17 provides an overview of the behavior of Q_meas1 in relation to P_meas1 and U_meas1. Notably, both trajectories in Fig. 17a,b exhibit the remarkable characteristic of returning to the exact starting point, indicating the capability of P_meas1 and Q_meas1 to effectively regain steady-state operation following the application of swells in both cases 5 and 6. Additionally, the trajectory of Q_meas1/U_meas1 in cases 5 and 6 (shown in Fig. 17c,d respectively) shows a closed path, affirming the proper restoration of U_meas1 to its initial conditions in both cases.

**Figure 18:**
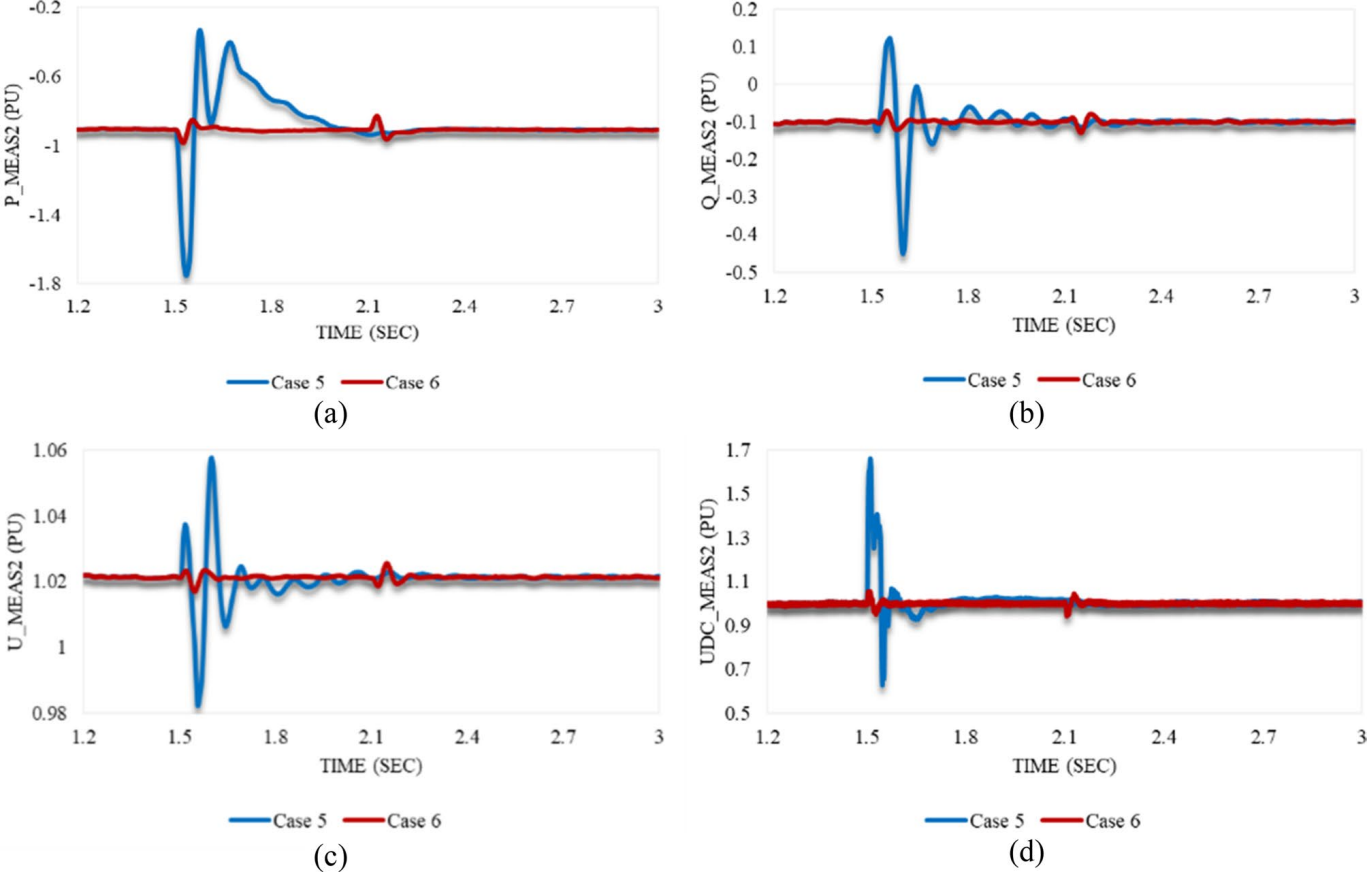
The measured waveforms at VSC_2_ when a stable swell disturbance is applied to the model at 1.5 s in both cases 5 and 6: (**a**) the active power, (**b**) the reactive power, (**c**) the RMS AC voltage, and (**d**) the DC voltage.

The behavior of P_meas2 due to the applied disturbance in cases 5 and 6 is shown in Fig. 18a. The delivered active power by VSC_2_ is excessively increased due to the sudden rise of the AC voltage in case 5. P_meas2 sharply declines to a significant US of 92.7% simultaneously with the applied disturbance. Then, it oscillates till stabilizing with a negligible SSE. In contrast, it initially decreases when the system is subjected to the disturbance in case 6, then slightly grows before reaching the steady state for a while. At 2.1 s, it oscillates again before regaining the steady-state condition. Q_meas2 follows up the Q_meas1 behavior in both cases 5 and 6 as illustrated in Fig. 18b. In case 5, Q_meas2 responds with a gradual increase followed by a sharp reduction with the US of 351%. It then oscillates until the steady-state conditions are reached. On the other hand, it slightly oscillates in case 6. According to Fig. 18c, U_meas2 oscillates similarly in both cases 5 and 6 except for the higher frequency of case 5 than case 6. Despite being impacted by the applied swell at VSC_1_, U_meas2 can regain stability properly. Figure 18d illustrates how Udc_meas2 responds to the applied disturbances in both cases 5 and 6. As can be seen from Figs. 16d and 18d, the DC voltage at both stations responds equally as usual in either cases 5 or 6.

**Figure 19:**
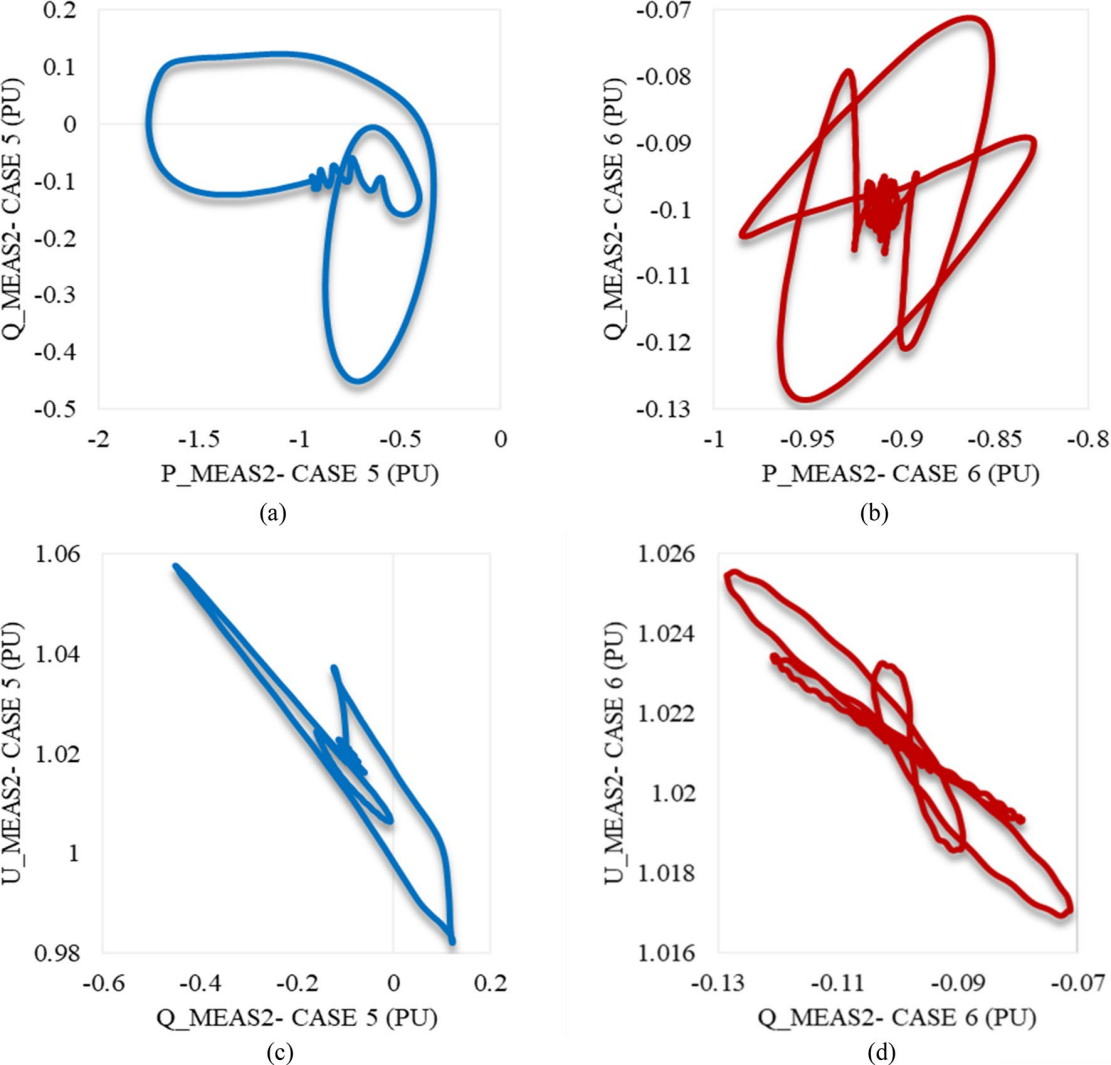
Trajectories of (**a**) active/reactive power at VSC_2_ in case 5, (**b**) active/reactive power at VSC_2_ in case 6, (**c**) reactive power/RMS AC voltage at VSC_2_ in case 5, and (**d**) reactive power/RMS AC voltage at VSC_2_ in case 6.

Figure 19a,b describe the trajectory of P_meas2/Q_meas2 clarifying their responses in both cases 5 and 6 respectively. The trajectory of Q_meas2/U_meas2 in cases 5 and 6 is illustrated in Fig. 19c,d respectively. As can be seen from Fig. 19, the closed paths of trajectories refer to the capability of VSC2 to regain stability if the system is subjected suddenly to the maximum swell limits (0.8 pu) for 2 cycles based on a system frequency of 50 Hz. In addition, it is also able to recover the steady state in the case of being applied to only 0.1 pu swell and lasts for the maximum duration of the instantaneous disturbance (30 cycles based on a frequency of 50 Hz).

### Impacting the model with unstable swell disturbances: Case 7 & Case 8

**Figure 20:**
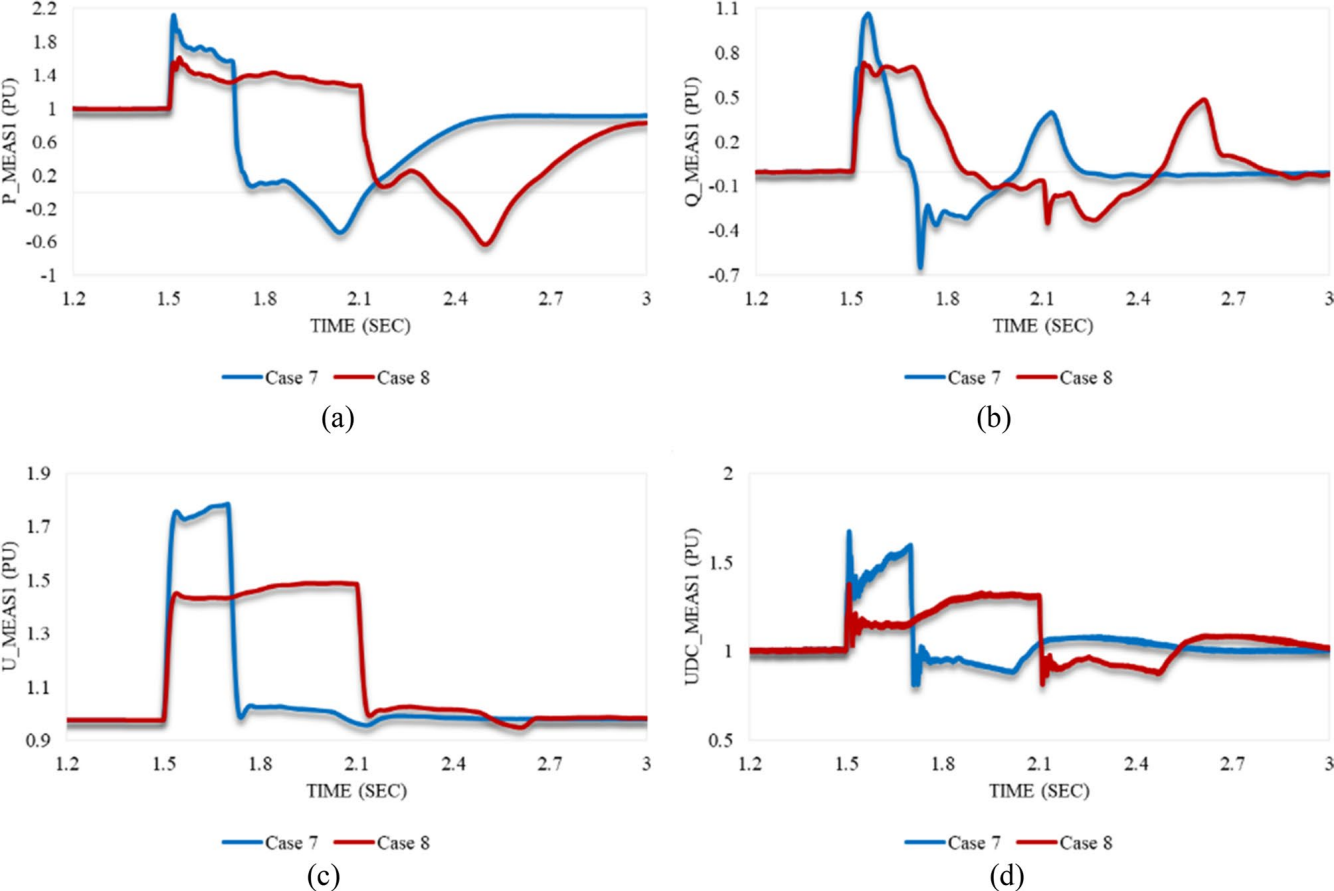
Measured waveforms at VSC1 when an unstable swell disturbance is applied to the model at 1.5 s in both cases 7 and 8: (**a**) the active power, (**b**) the reactive power, (**c**) the RMS AC voltage, and (**d**) the DC voltage.

Figure 20a depicts that P_meas1 behaves in both cases 7 and 8 in the same way. It grows simultaneously with the instant at which the disturbance is applied to the system. After the disturbance clearance, it falls to a significant US% before a gradual rise until steady state conditions are attained. Despite the greater US% of P_meas1 in case 7 than in case 8, it regains stability quicker with a smaller SSE in case 7 compared to case 8. As shown in Fig. 20b, Q_meas1 responds to both disturbances in cases 7 and 8 by oscillating, followed by a proper regain of system stability. The system instability can be easily noticed from the reactive power response in case of applying an unstable swell disturbance to one of the converter stations. On the other hand, active power is an indicator of system instability when the system is subjected to an unstable sag disturbance.

**Figure 21:**
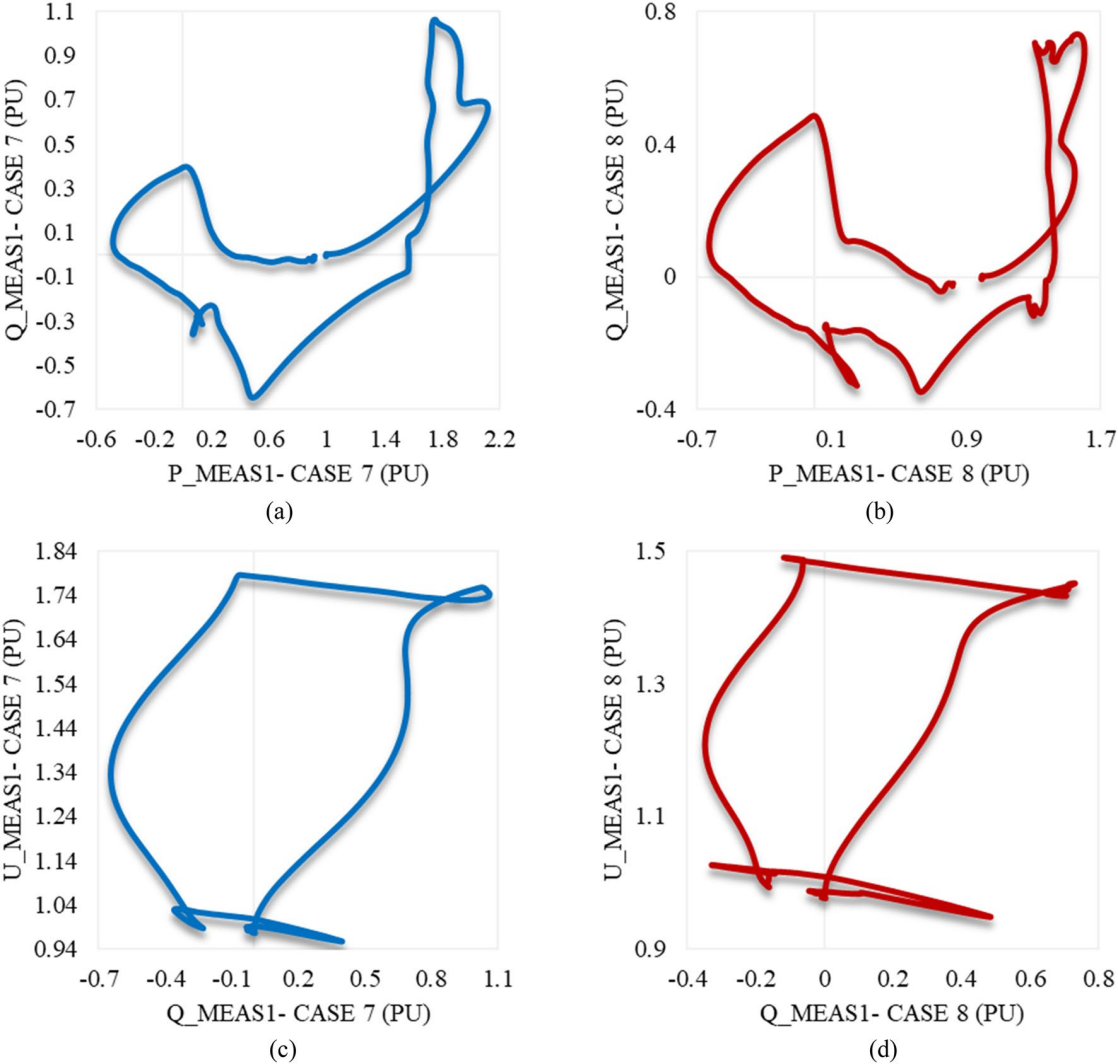
Trajectories of (**a**) active/reactive power at VSC_1_ in case 7, (**b**) active/reactive power at VSC_1_ in case 8, (**c**) reactive power/RMS AC voltage at VSC_1_ in case 7, and (**d**) reactive power/RMS AC voltage at VSC_1_ in case 8.

U_meas1 rises significantly in both cases 7 and 8 in the same way as depicted in Fig. 20c. It increases, then temporarily settles before recapturing its stability. Although the OS% of U_meas1 in case 7 is greater than case 8, it attains the steady state conditions in case 7 quicker than case 8. Figure 20d reveals that the DC voltage at VSC_1_ initially grows for a while before a steep reduction is noticed in either case 7 or 8. Then, it gradually regains its stability. The trajectory of P_meas1/Q_meas1 that describes their responses to cases 7 and 8 corresponding to each other is shown in Fig. 21a,b respectively. As both paths are open, that indicates the disability of station 1 to recapture stability when the disturbances of cases 7 and 8 are applied to the system.

**Figure 22:**
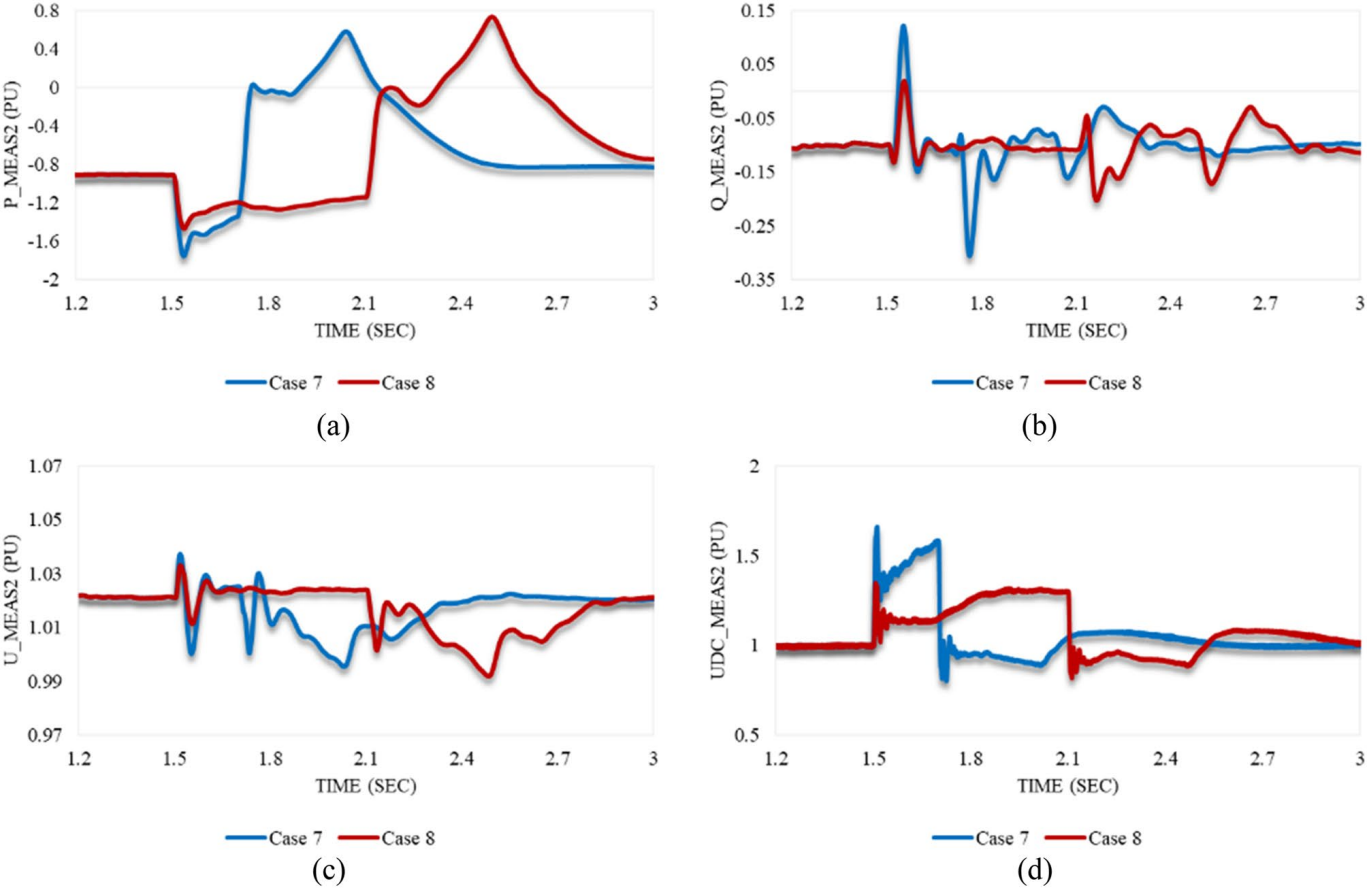
Measured waveforms at VSC_2_ when an unstable swell disturbance is applied to the model at 1.5 s in both cases 7 and 8: (**a**) the active power, (**b**) the reactive power, (**c**) the RMS AC voltage, and (**d**) the DC voltage.

**Figure 23:**
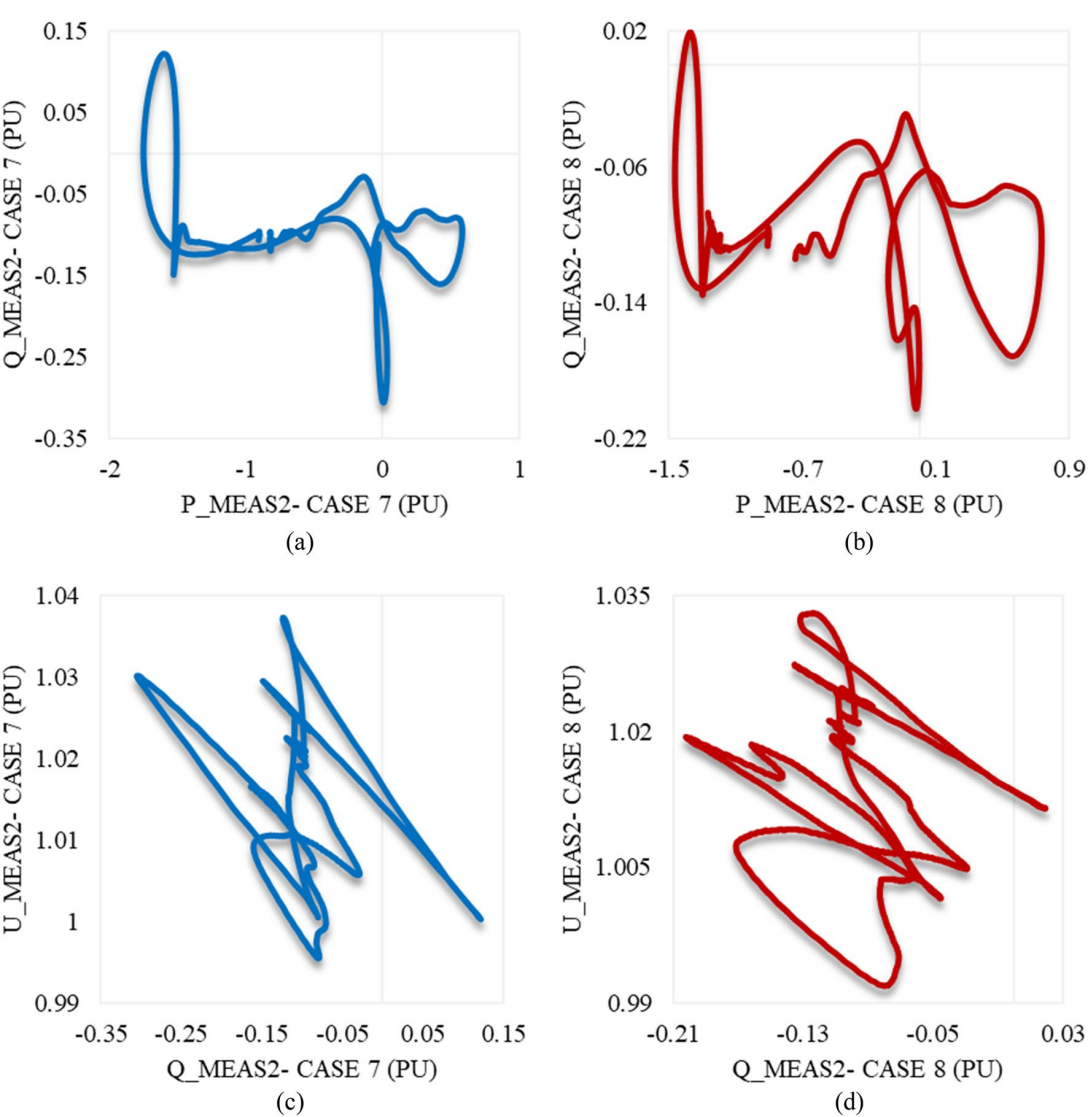
Trajectories of (**a**) active/reactive power at VSC_2_ in case 7, (**b**) active/reactive power at VSC_2_ in case 8, (**c**) reactive power/RMS AC voltage at VSC_2_ for case 7, and (**d**) reactive power/RMS AC voltage at VSC_2_ in case 8.

Figure 22a illustrates the behavior of P_meas2 due to applied disturbances in cases 7 and 8 to the system. As can be seen, it counteracts the response of P_meas1 in either case 7 or 8 by receiving the transmitted power from VSC_1_, then gradually declines until the steady state is regained with a negligible SSE. Figure 22b,c show how Q_meas2 and U_meas2 respond to the disturbances in both cases 7 and 8 respectively. The reactive power at both converter stations behaves equally due to these disturbances see Figs. 20b and 22b. It oscillates till the steady-state conditions are reached. However, Q_meas2 oscillates with a higher frequency than Q_meas1. In contrast to U_meas1, U_meas2 sharply oscillates before a significant reduction is noticed, as can be seen from Figs. 20c and 22c. The DC voltage at both converter stations responds identically when the system is subjected to the disturbance of either case 7 or 8 (see Figs. 20d and 22d. Figure 23 depicts the trajectories of P_meas2/Q_meas2 and Q_meas2/U_meas2, revealing that VSC2 fails to recover its steady-state operation when subjected to system disturbances in both cases 7 and 8.

## Future perspectives

Table 3 provides a comprehensive comparison concerning the applied methodology in this research against the recent research studies. Thus, future research can focus on studying VSC-HVDC transmission systems under imposed power quality challenges. This can be applied by implementing optimization technique in a real-world VSC-HVDC transmission system and evaluate its performance under various operational conditions. Furthermore, methods for maintaining network voltage stability under these disturbances should be developed. By addressing these power quality challenges, the performance and reliability of VSC-HVDC systems can be improved. Future research can also be enhanced through exploring the impact of adjusting some parameters like the DC line length as well as capacitor bank size on the conducted case studies.

**Table 3:** Competitive comparison of recent studies’ methodologies.

Point of comparison	Available methodologies	Features	References
VSC technology	MMC	Provides different voltage levels	^39,45–49^
	NPC	Widely used in industrial applications Negligible harmonics Minimized losses	This study
Prevalent fields of study	Multi-terminal systems	Integration of RES to passive networks	^39,50,51^
		Super Grids Asynchronous AC Systems Interconnection Bidirectional Power Flow	This study
	Voltage sag	Causes in VSC-HVDC System Impacts on VSC-HVDC System Critical Values Derivation like CV and CCT Performance Analysis under Unstable Sags	This study
	Voltage swell	Causes in VSC-HVDC System Impacts on VSC-HVDC System Critical Values Derivation like CV and CCT Performance Analysis under Unstable Swells	This study

## Conclusion

This paper studies two asynchronous AC sources that are interconnected through a VSC-HVDC transmission system comprising three arms-NPC converters. The system is simulated using MATLAB/Simulink. The frequent PQ phenomena, namely voltage sag and swell, are applied to the model by manipulating the AC voltage of the controllable voltage source at VSC_1_. Active/reactive power and AC/DC voltage at both converter stations are recorded and studied. The obtained results demonstrate the proper bidirectional power transmission through the VSC-HVDC system as well as the fast recovery after being subjected to instant voltage sag and swell. The active/reactive power and the RMS AC voltage of both converter stations counteract each other's responses to meet the power flow requirements. For instance, the applied sag disturbance at VSC_1_ forces the transmitted active power to be reversed from VSC_2_ to VSC_1,_ unlike the initial operational conditions. As a result, the active power always decays at VSC_1_ and grows at VSC_2_. Since the voltage sag reduces the RMS AC voltage at VSC_1_, it consequently lessens the required reactive power at VSC_1_. However, the reactive power as well as the RMS AC voltage at VSC_2_ increase as it becomes a temporary sending end. The system barely remains robust during the instant sag disturbance for a brief time. It withstands the maximum sag limit (0.9 pu) for only 160 ms. In contrast to the sag impact on the studied model, the voltage swell issue results in a significant rise in the RMS AC voltage at VSC_1_. Hence, the generated reactive power at this converter station follows this sudden change. In addition, the active power is drawn from VSC_1_ to VSC_2_, as expected. However, the system hardly sticks to the stable operational conditions during the maximum swell perturbation (0.8 pu) for only 40 ms. Regardless of whether a sag or swell disturbance is applied to the model, the DC voltage at both stations exhibits consistent fluctuations. This finding highlights the importance of activating the DC voltage balance control to minimize these oscillations. Thus, it ensures a more stable and controlled operation of the system. This control also plays a crucial role in maintaining the integrity and reliability of the converter stations, contributing to the system’s efficient performance.

## Data Availability

The datasets used and/or analyzed during the current study are available from the corresponding author upon request.

## References

[1] Hairong, C., Fan, Z. & Yong, C. Improvement of power quality by VSC based multi-terminal HVDC. In *2006 IEEE Power Eng. Soc. Gen. Meet. PES* 1–6 (2006). 10.1109/pes.2006.1709257.

[2] Kumar R, Singh B, Shahani DT (2016). Symmetrical components-based modified technique for power-quality disturbances detection and classification. IEEE Trans. Ind. Appl..

[3] Yu M, Dysko A, Booth CD, Roscoe AJ, Zhu J (2014). A review of control methods for providing frequency response in VSC-HVDC transmission systems. Proc. Univ. Power Eng. Conf..

[4] Tang X, Lu DDC (2014). Enhancement of voltage quality in a passive network supplied by a VSC-HVDC transmission under disturbances. Int. J. Electr. Power Energy Syst..

[5] Zhu J (2022). Inertia Emulation and fast frequency-droop control strategy of a point-to-point VSC-HVdc transmission system for asynchronous grid interconnection. IEEE Trans. Power Electron..

[6] Wang, H. & Redfern, M. A. Enhancing AC networks with HVDC interconnections. In *CICED 2010 Proceedings* 1–7 (2010).

[7] Ajaei FB, Iravani R (2016). Dynamic interactions of the MMC-HVDC grid and its host AC system due to AC-side disturbances. IEEE Trans. Power Deliv..

[8] Navpreet T (2012). Voltage Source Converters as the building block of HVDC and FACTS technology in power transmission system: A simulation based approach. Pelagia Res. Libr. Adv. Appl. Sci. Res..

[9] Kangwa, N. M., Venugopal, C. & Davidson, I. E. A review of the performance of VSC-HVDC and MTDC systems. In *Proc. - 2017 IEEE PES-IAS PowerAfrica Conf. Harnessing Energy, Inf. Commun. Technol. Afford. Electrif. Africa, PowerAfrica 2017*, 267–273 (2017). 10.1109/PowerAfrica.2017.7991235.

[10] Du, C. & Agneholm, E. A novel control of VSC-HVDC for improving power quality of an industrial plant. In *IECON Proc. (Industrial Electron. Conf.* 1962–1967 (2006). 10.1109/IECON.2006.347538.

[11] He X, Wang R, Wu J, Li W (2020). Nature of power electronics and integration of power conversion with communication for talkative power. Nat. Commun..

[12] Li Y, Zhu G, Zhou K, Meng P, Wang G (2021). Evaluation of graphene/crosslinked polyethylene for potential high voltage direct current cable insulation applications. Sci. Rep..

[13] Jayachandran M, Reddy CR, Padmanaban S, Milyani AH (2021). Operational planning steps in smart electric power delivery system. Sci. Rep..

[14] Van Hertem D, Ghandhari M (2010). Multi-terminal VSC HVDC for the European supergrid: Obstacles. Renew. Sustain. Energy Rev..

[15] Zhang L, Harnefors L, Nee HP (2011). Modeling and control of VSC-HVDC links connected to island systems. IEEE Trans. Power Syst..

[16] Wei L (2012). Voltage source converter based HVDC. Energy Procedia.

[17] Oni OE, Mbangula KI, Davidson IE (2016). A review of LCC-HVDC and VSC-HVDC technologies and applications. Trans. Environ. Electr. Eng..

[18] Xiao H, Sun K, Pan J, Li Y, Liu Y (2021). Review of hybrid HVDC systems combining line communicated converter and voltage source converter. Int. J. Electr. Power Energy Syst..

[19] Huang H, An F (2022). Review on MMC-HVDC and Its Power Quality.

[20] Stan A, Costinaș S, Ion G (2022). Overview and assessment of HVDC current applications and future trends. Energies.

[21] Flourentzou N, Agelidis VG, Demetriades GD (2009). VSC-based HVDC power transmission systems: An overview. IEEE Trans. Power Electron..

[22] Bollen, M. H. J., Bahramirad, S. & Khodaei, A. Is there a place for power quality in the smart grid? In *Proc. Int. Conf. Harmon. Qual. Power, ICHQP*, 713–717 (2014). 10.1109/ICHQP.2014.6842865.

[23] Bash, E. *Hvdc Grids-Book*. Ph.D. Proposal, vol. 1 (2015).

[24] Wang H, Ma KW (2016). IGBT technology for future high-power VSC-HVDC applications. IET Conf. Publ..

[25] IEEE Std. *IEEE Recommended Practice for Monitoring Electric Power Quality*. *IEEE Std 1159 - 1995* vol. 2019 (1995).

[26] Augustin, T., Jahn, I., Norrga, S. & Nee, H. P. Transient behaviour of VSC-HVDC links with DC breakers under faults. In *2017 19th Eur. Conf. Power Electron. Appl. EPE 2017 ECCE Eur.* 2017-Janua, 1–10 (2017).

[27] Sabin, D., Norwalk, M., Kittredge, K. & Johnston, S. IEEE Power Quality Standards. In *2022 20th International Conference on Harmonics & Quality of Power (ICHQP)*, 1–6 (2022). 10.1109/ICHQP53011.2022.9808543.

[28] Prasad, M. & Akella, A. K. Voltage sag characteristics in power distribution system under fault conditions. *Energy Educ. Sci. Technol. Part A Energy Sci. Res.* **33**(6), 3177–3192 (2015)

[29] Han Y (2020). Cause, classification of voltage sag, and voltage sag emulators and applications: A comprehensive overview. IEEE Access.

[30] Andrei, H. *et al.* Electrical power systems. In *Power Systems*, 3–47 (2017). 10.1007/978-3-319-51118-4_1.

[31] Egea-Alvarez A, Barker C, Hassan F, Gomis-Bellmunt O (2015). Capability curves of a VSC-HVDC connected to a weak AC grid considering stability and power limits. IET Semin. Dig..

[32] Li B, Shi S, Xu D, Wang W (2016). Control and analysis of the modular multilevel dc de-icer with statcom functionality. IEEE Trans. Ind. Electron..

[33] Zhang, X., Yao, L., Chong, B., Sasse, C. & Godfrey, K. R. Development Technologies for the of Future Power Systems. In *2005 Int. Conf. Futur. Power Syst.* 4–9.

[34] Nam, T., Shim, J. W. & Hur, K. Design and operation of double SMES coils for variable power system through VSC-HVDC connections. *IEEE Trans. Appl. Supercond.* **23** (2013).

[35] Biswas, A. K., Ahmed, S. I., Akula, S. K. & Salehfar, H. High voltage AC (HVAC) and high voltage DC (HVDC) transmission topologies of offshore wind power and reliability analysis. In *IEEE Green Technol. Conf.* **2021**-**April**, 271–278 (2021).

[36] Ryndzionek R, Sienkiewicz Ł (2020). Evolution of the HVDC link connecting offshore wind farms to onshore power systems. Energies.

[37] Yazdani A, Iravani R (2006). Dynamic model and control of the NPC-based Back-to-Back HVDC system. IEEE Trans. Power Deliv..

[38] Khatir M, Zidi SA, Hadjeri S, Fellah MK (2010). Dynamic performance of a back-to-back HVDC station based on voltage source converters. J. Electr. Eng..

[39] Hannan MA (2018). Advanced control strategies of VSC based HVDC transmission system: Issues and potential recommendations. IEEE Access.

[40] Yang, Z., Dao, Z. & Yu, F. The development of HVDC transmission system. In *Proc. - 2012 3rd Int. Conf. Digit. Manuf. Autom. ICDMA 2012* 907–910 (2012). 10.1109/ICDMA.2012.214.

[41] Khillo, A. & Patnaik, S. S. Performance Analysis of 6-Pulse HVDC-VSC using particle swarm optimization (PSO) based controller in d-q reference frame under transient AC fault conditions. In *2020 5th IEEE Int. Conf. Recent Adv. Innov. Eng. ICRAIE 2020—Proceeding* 1–6 (2020). 10.1109/ICRAIE51050.2020.9358345.

[42] Shah R, Sánchez JC, Preece R, Barnes M (2018). Stability and control of mixed AC–DC systems with VSC-HVDC: A review. IET Gener. Transm. Distrib..

[43] Khosravi N (2022). Improvement of power quality parameters using modulated-unified power quality conditioner and switched-inductor boost converter by the optimization techniques for a hybrid AC/DC microgrid. Sci. Rep..

[44] Eyenubo O, Oshevire P (2017). Improvement of power system quality using VSC-based HVDC transmission. Niger. J. Technol..

[45] Yanwei W, Jiajun O, Tao S, Bo W, Junming W (2023). VSC control strategy for HVDC compensating negative sequence and decaying DC components. Energy Rep..

[46] Raziq H (2023). Power quality improvement of a distribution system integrating a large scale solar farm using hybrid modular multilevel converter with ZSV control. Ain Shams Eng. J..

[47] Tiwari RS, Kumar R, Gupta OH, Sood VK (2023). Dynamic analysis of VSC-HVDC system with disturbances in the adjacent AC networks. Distrib. Gener. Altern. Energy J..

[48] Li W, Liu R, Li Y (2023). Power quality enhancement of remote gas field generations with smart power converters. Energies.

[49] Maysse IE (2023). Nonlinear observer-based controller design for VSC-based HVDC transmission systems under uncertainties. IEEE Access.

[50] Lin C-H, Wu Y-K (2023). Coordinated frequency control strategy for VSC-HVDC-connected wind farm and battery energy storage system. IEEE Trans. Ind. Appl..

[51] Lu Z, Ye Y, Qiao Y (2019). An adaptive frequency regulation method with grid-friendly restoration for VSC-HVDC integrated offshore wind farms. IEEE Trans. Power Syst..

